# GeauxDock: Accelerating Structure-Based Virtual Screening with Heterogeneous Computing

**DOI:** 10.1371/journal.pone.0158898

**Published:** 2016-07-15

**Authors:** Ye Fang, Yun Ding, Wei P. Feinstein, David M. Koppelman, Juana Moreno, Mark Jarrell, J. Ramanujam, Michal Brylinski

**Affiliations:** 1 School of Electrical Engineering and Computer Science, Louisiana State University, Baton Rouge, Louisiana, United States of America; 2 Department of Physics and Astronomy, Louisiana State University, Baton Rouge, Louisiana, United States of America; 3 High-Performance Computing, Louisiana State University, Baton Rouge, Louisiana, United States of America; 4 Department of Biological Sciences, Louisiana State University, Baton Rouge, Louisiana, United States of America; 5 Center for Computation & Technology, Louisiana State University, Baton Rouge, Louisiana, United States of America; UMR-S1134, INSERM, Université Paris Diderot, INTS, FRANCE

## Abstract

Computational modeling of drug binding to proteins is an integral component of direct drug design. Particularly, structure-based virtual screening is often used to perform large-scale modeling of putative associations between small organic molecules and their pharmacologically relevant protein targets. Because of a large number of drug candidates to be evaluated, an accurate and fast docking engine is a critical element of virtual screening. Consequently, highly optimized docking codes are of paramount importance for the effectiveness of virtual screening methods. In this communication, we describe the implementation, tuning and performance characteristics of GeauxDock, a recently developed molecular docking program. GeauxDock is built upon the Monte Carlo algorithm and features a novel scoring function combining physics-based energy terms with statistical and knowledge-based potentials. Developed specifically for heterogeneous computing platforms, the current version of GeauxDock can be deployed on modern, multi-core Central Processing Units (CPUs) as well as massively parallel accelerators, Intel Xeon Phi and NVIDIA Graphics Processing Unit (GPU). First, we carried out a thorough performance tuning of the high-level framework and the docking kernel to produce a fast serial code, which was then ported to shared-memory multi-core CPUs yielding a near-ideal scaling. Further, using Xeon Phi gives 1.9× performance improvement over a dual 10-core Xeon CPU, whereas the best GPU accelerator, GeForce GTX 980, achieves a speedup as high as 3.5×. On that account, GeauxDock can take advantage of modern heterogeneous architectures to considerably accelerate structure-based virtual screening applications. GeauxDock is open-sourced and publicly available at www.brylinski.org/geauxdock and https://figshare.com/articles/geauxdock_tar_gz/3205249.

## Introduction

The goal of drug discovery is to identify, optimize and clinically validate those compounds that bind and modulate the function of a target protein implicated in a disease state. A drug molecule must possess certain geometry and physicochemical properties in order to have a sufficiently high binding affinity toward a given macromolecular target. As a result, the number of bioactive compounds is very small compared to a vast collection of candidate compounds. For example, the ZINC database of commercially available small molecule entities consists of 17,900,742 drug-like compounds collected from 243 vendors as of January 2016 [1]. Considering molecules yet to be synthesized, the chemical universe comprises an estimated novemdecillion (10^60^) of small organic compounds [2]. At the outset of drug discovery, this large number of candidates need to be downsized to hundreds or thousands of the most promising compounds. Experimental high-throughput screening is a conventional approach used by the pharmaceutical industry to identify bioactive molecules, however, it suffers from high costs and relatively low hit rates [3]. For instance, a recent study by the Tufts Center for the Study of Drug Development estimates that the development of a new prescription medicine typically continues for longer than a decade with the total costs of over 2.5 billion US dollars [4]. Not surprisingly, modern drug discovery is increasingly supported by computational modeling to reduce the overall costs, improve the efficiency and speed up the development time. As an example, a fast drug development is critical in combating the Ebola virus, therefore, computational approaches are expected to significantly contribute to Ebola research through protein structure modeling and large-scale docking of small molecule libraries against viral proteins [5].

One of the most widely used techniques for ligand virtual screening is structure-based molecular docking to model the binding pose of a ligand in the binding site of the receptor protein followed by the prediction of binding affinity and/or free energy [6]. In contrast to ligand-based approaches that need an initial set of bioactive compounds, the only experimental data required for structure-based docking is the 3D structure of the protein target, although homology models can be used instead [7,8]. Consequently, these methods are well positioned to take advantage of the continuously growing structure databases, such as the Protein Data Bank (PDB) [9], providing opportunities to discover novel biopharmaceuticals. Because of the importance of ligand docking in modern drug development, a number of programs have been developed to date [10]. In general, using large compound databases increases the chances of finding bioactives, however, large-scale virtual screening typically requires a long computing time. In addition to the database size, computing time also increases with the increasing accuracy of the modeling of drug-protein interactions. Although sophisticated models outperform simple approaches, these algorithms often have high demands for computational resources. For example, docking accuracy can be improved by incorporating the plasticity of biomolecules, e.g. using pre-generated ensembles of target protein structures [11]. Since ensemble-based docking requires conducting docking simulation for each target conformation, the computational complexity increases linearly with the number of conformers. Another approach to improve ligand docking incorporates the configurational entropy. This property can be approximated by clustering ligand binding poses generated by a docking program to calculate the conformational similarity between each pair of ligand modes, leading to O(n^2^) complexity, where *n* is the total number of binding poses. Mining Minima provides a more accurate way to calculate entropy by integrating potential energies as a function of coordinates, however, at a significantly increased computational cost [12]. Finally, the simulation time can also affect docking accuracy for those docking programs relying on stochastic methods to sample the free energy landscape, where longer simulations are more likely to reach the global minimum [13].

Undeniably, achieving a good balance between docking accuracy and the computation time represents a major challenge in structure-based virtual screening. To address this problem, parallel computing is often used to accelerate docking simulations. Parallel architectures fall into two broad categories: 1) small groups of tightly coupled processors sharing a common memory space, and 2) large, scalable systems that do not share a common memory. Both models often coexist in a high-performance computing (HPC) environment; for instance, many HPC systems use the distributed memory model to scale up to thousands of multi-processor nodes, each employing the shared memory model. A common programming practice for shared memory systems is to inform the compiler of parts of the serial code to be executed in parallel by including extra hints, e.g. using OpenMP pragmas [14]. In contrast, distributed memory systems require manually implemented message-passing procedures, e.g. using Message Passing Interface (MPI) protocols [15]. Parallel programming used to be a small niche until the traditional single-core Central Processing Unit (CPU) hit the "instruction level parallelism wall” and the "clock speed wall" [16] a decade ago. Although CPU vendors managed to bypass these limitations by integrating more computing cores into a CPU, contemporary multi-core CPUs are not the ultimate solution due to the power [17] and energy [18] problems. A new trend in processor design to replace a handful of heavyweight cores with a massive amount of lightweight computing units upthrust parallel programming to the mainstream.

In contrast to traditional CPU architectures designed to minimize the execution latency of serial codes, highly simplified cores of modern accelerators are generally optimized for high-throughput computations, therefore, their performance on latency-sensitive applications is often poor. Consequently, these computing units are usually attached to conventional CPU-based systems as heterogeneous devices equipped with their own memory. Two major accelerator architectures currently available, NVIDIA Graphics Processing Unit (GPU) and Intel Xeon Phi, share some common features, but also have unique characteristics. With respect to hardware, both accelerators as well as contemporary multi-core CPUs share a two-level parallel design principle. The outer, coarse-grained level defines a computation cluster whose individual processing elements provide the inner, fine-grained level of parallelism. With regard to software, each coarse-grained cluster handles its own programming context known as a thread on CPU and Xeon Phi, and a thread block defined by the GPU Compute Unified Device Architecture (CUDA) [19] paradigm. On CPU and Xeon Phi, the inner level exposes data parallelism, *viz*. Single Instruction, Multiple Data (SIMD) operations. NVIDIA GPU uses CUDA threads inheriting a similar principle of vector processing. For instance, a bundle of 32 consecutive CUDA threads, denoted as a warp, are scheduled together. Consequently, CUDA threads may go predication when a small, conditionally protected piece of code is encountered, forcing the execution of all instructions.

When different CUDA threads take different paths in multiple-path branches, more cycles are consumed leading to a lower device utilization. Although SIMD instructions on CPU and Xeon Phi have similar characteristics, the number of vector elements is about one-quarter to one-half of that on GPU and the code generation heuristic can vary significantly, therefore, an irregular code may perform dramatically differently on these platforms. Another major difference between CPU and Xeon Phi, and GPU is that the former implement hardware multi-threading at the outer level, whereas multi-threading on GPU is at the inner level demanding more data parallelism. Compared to CPU, contemporary Xeon Phi delivers roughly equal amount of raw compute power per core in terms of the number of data operations per cycle. However, because of a larger number of computing cores on the co-processor, it offers certain advantages over CPU in processing regular, highly parallel workloads. On the other hand, CPU cores typically perform better for irregular workloads. In addition to core characteristics, computing performance is also affected by memory operations. Different from the automatic memory management as cache on CPU and Xeon Phi, GPU exposes to programmers its fast on-chip memory, known as the CUDA shared memory.

A common programming practice for GPU is to exploit the parallelism using low-level Application Programming Interfaces (APIs), such as CUDA and OpenCL [20]. GPU programming typically comprises several stages, 1) identify parallel workloads, 2) copy data from the host to the device, 3) map workloads to computing cores, 4) determine a suitable memory access for CUDA threads, 5) synchronize the execution between GPU and CPU, and 6) copy data back to the host. Despite a significant effort directed to help automate these steps, high-level GPU programming languages are still not versatile enough to fully unleash the power of GPU for complex applications. In contrast, Xeon Phi is designed to provide massive parallelism at considerably reduced programming efforts. Intel compilers can generate Xeon Phi accelerated binaries in a similar way to compiling traditional CPU codes [21], therefore, programming Xeon Phi in the native mode is fairly comparable to coding for multiple-core CPUs. Similar to GPU, Xeon Phi also offers an offload mode, where only selected portions of the code marked by compiler pragmas are executed on the accelerator. OpenMP can be used in both native and offload modes alleviating the need for low-level implementations.

In order to address computational challenges in structure-based virtual screening, several docking programs offer HPC capabilities. For instance, AutoDock Vina [22] supports multi-threading on CPU using the Boost::thread library yielding significant speedups on multi-core processors compared to a serial version. Moreover, a CUDA implementation of MolDock accelerates both the evolution search algorithm and its two-element scoring functions on GPU [23], whereas PLANTS employs a systematic grid search with an accelerated scoring function on GPU using a high-level shading language [24]. A few projects take the heterogeneous concept one step further by developing a hybrid docking framework that can be executed on different computer architectures. For example, non-bonded interactions in molecular dynamics kernels were parallelized for both GPU (using CUDA) and CPU (using OpenMP), and further extended to fully utilize distributed platforms through MPI protocols [25]. The docking engine BUDE [26] employs the OpenCL language to maintain a parallel implementation of the genetic search algorithm for CPU, Xeon Phi and GPU. Nonetheless, to the best of our knowledge, an efficient multiple-backend implementation of the docking kernel based on Metropolis Monte Carlo (MMC) has not been reported yet.

Recently, we developed GeauxDock, a new molecular docking package to model drug-protein complexes using a mixed-resolution molecular representation and the MMC search engine [27]. GeauxDock uses non-hydrogen atoms for ligands, whereas proteins are described at the coarse-grained, sub-residual level. Such a mixed-resolution description not only helps tolerate structural deformations in the target binding sites caused by using protein models as docking targets, but also speeds up calculations by decreasing the number of interaction points on macromolecules. The descriptor-based force field implemented in GeauxDock includes nine energy terms carefully optimized to drive docking simulations toward native-like conformations using a multi-replica MMC sampling. Furthermore, GeauxDock employs an ensemble-based approach to effectively model the flexibility of ligands and proteins. Although GeauxDock simulations typically converge in less than 1,000 MMC cycles on standard datasets, its large-scale virtual screening applications remain computationally challenging due to a large number of candidate molecules to be evaluated. On that account, the present study describes our efforts porting GeauxDock to multi-core CPUs and massively parallel accelerators, Xeon Phi and GPU. Computational models and performance patterns are analyzed in detail for different architectures. We also discuss various code characteristics as well as general and platform-specific optimization techniques used to turn GeauxDock into an ultra-fast docking tool for large-scale drug virtual screening.

## Materials and Methods

### Virtual screening workflow

**Fig 1:**
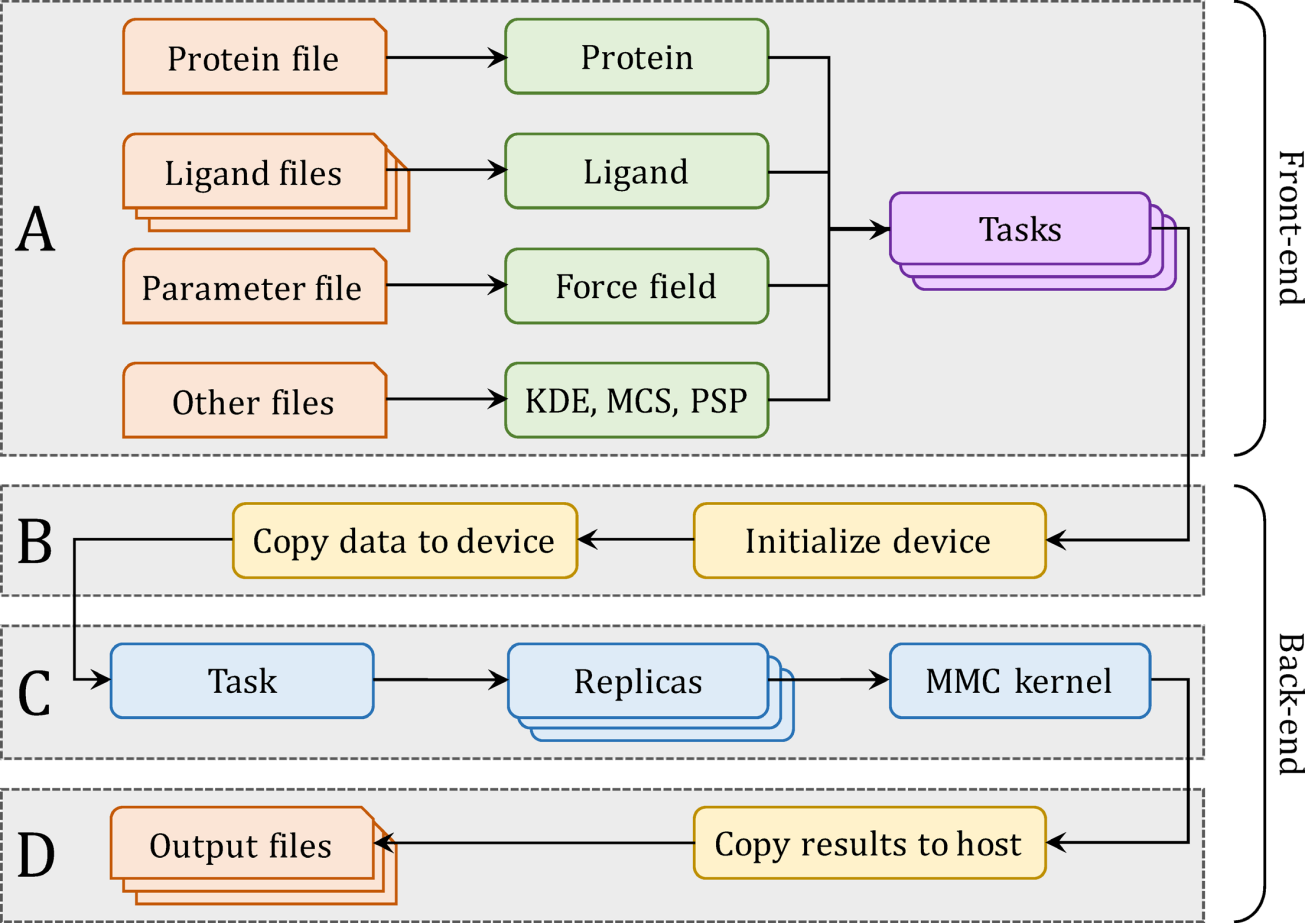
Workflow of virtual screening using GeauxDock. (**A**) The front-end reads input data and creates a pool of docking tasks. The back-end carries out three consecutive operations: (**B**) device initialization and data transfer, (**C**) docking calculations for individual tasks, and (**D**) saving output data.

GeauxDock is designed for virtual screening applications, where a given protein target is screened against a large library of small organic compounds. A docking simulation of a single ligand is an independent computational task. Fig 1 shows four stages of virtual screening using GeauxDock. The procedure starts with reading the input data and creating a pool of tasks (Fig 1A). Protein and ligand files provide the initial coordinates of the target protein and library compounds. The parameter file specifies various parameters, such as coefficients to calculate energy terms, weight factors to linearly combine individual energy components, as well as the length of rotation and translation vectors to perturb ligand conformations during MMC simulations. Other files contain data to calculate a pseudo-pharmacophore using the Kernel Density Estimation (KDE), restraints on family-conserved anchor substructures using the Maximum Common Substructure (MCS), and a pocket-specific potential (PSP). The KDE component of the scoring function describes the likelihood of target ligand atoms to be at certain positions with respect to template-bound ligand atoms, whereas the MCS term imposes root-mean-square deviation (RMSD) restraints according to a chemical matching between the target ligand and template-bound ligands collected from the PDB [27,28]. Further, PSP is a contact-based statistical potential derived from weakly homologous holo-templates identified by threading rather than all protein-ligand complexes present in the PDB [27,29]. Once the required input data are read and pre-processed, a computing device is initialized and the data is copied to the accelerator (Fig 1B). Subsequently, docking calculations are performed for individual tasks (Fig 1C) and finally, the output files are generated on the host (Fig 1D).

Preliminary testing of this workflow reveals that the redundant loading and parsing of the same target protein when docking different ligands consumes up to 90% of the total I/O time (Table 1). As a consequence of these excessive I/O operations, the execution of MMC kernels on GPU makes for only 52% of the total simulation time. Furthermore, the repetitive GPU memory allocation and de-allocation performed for each task takes almost as much time as running the MMC kernel. Although the code for Xeon Phi is expected to have similar issues, the compiler pragmas are placed inside the MMC kernel code, thus the entire offload procedure combines data transfer and core calculations. The memory management for the code offload is not required in the CPU implementation. To address the problem of the excessive I/O operations particularly for GPU-based platforms, the four-step workflow for GeauxDock is arranged into two parts. The front-end consists of data loading, pre-processing and creating a pool of tasks (Fig 1A), whereas the back-end fetches tasks, initializes a computing device, executes the docking kernel, and periodically saves the output data (Fig 1B–1D). With this design, the memory allocation and de-allocation on GPU occur only once at the beginning and the end of the back-end process, respectively.

**Table 1:** Time in ms required to complete various stages of a docking simulation by GeauxDock for the 1a07 complex.

Computing platform	Loading data		Initialization		Simulation	Output generation		Total
	*Protein*	*Other*	*Device MemAlloc*	*Copy data to device*	*Docking kernel*	*Copy data from device*	*Device MemFree*	
CPU	214	21	-	-	4,848	-	-	2,740
Xeon Phi	214	21	3,135 (initialization + simulation + output)					3,374
GPU	216	21	2,063	0.72	2,740	8	182	5,237

Docking kernel performs 1,000 MMC cycles.

### Code implementation

**Fig 2:**
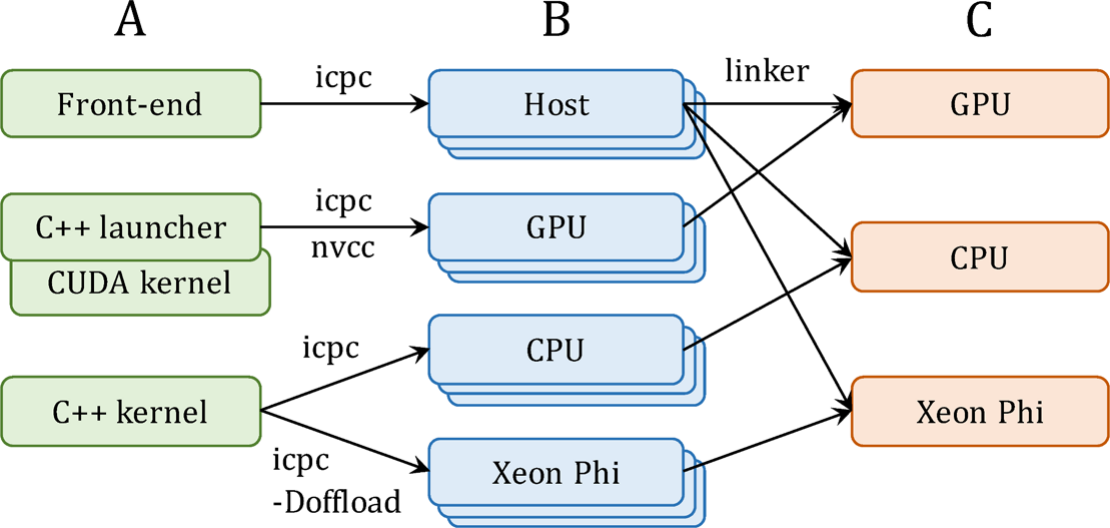
Implementation of GeauxDock. (**A**) The code repository is divided into three modules, a common front-end module for the CPU host and two back-end modules, one for GPU and one for CPU and Xeon Phi. (**B**) Compiling the source codes produces a series of architecture-specific object files. (**C**) Linking object files creates three binary versions for GPU, CPU and Xeon Phi.

Docking simulations with GeauxDock can be conducted on three platforms, multi-core CPU, GPU and Xeon Phi. Therefore, the source code is modularized for an easy maintenance across different architectures (Fig 2). All three platforms share a common code for front-end computations, whereas back-end codes have two versions, one for CPU and Xeon Phi, and one for GPU. The C++ kernel employing OpenMP and Intel SIMD pragmas is shared between CPU and Xeon Phi. Using the “-Doffload” flag enables additional pragmas protected by the “#ifdef offload” macro, which instruct the compiler to generate object files for Xeon Phi instead of CPU. In contrast, the GPU version comprises a C++ launcher and a docking kernel implemented in CUDA. This design allows for maintaining a single front-end code and two versions of the back-end code. Compiling the source codes (Fig 2A) generates architecture-specific object files (Fig 2B), which are linked to create different versions of the binary (Fig 2C).

### Parallelization levels

GeauxDock features an enormous task-level parallelism, where different library compounds docked against the target protein correspond to individual tasks. In addition, the docking kernel exploits coarse- and fine-grained parallelism. Docking calculations for a single task involve multiple protein and ligand conformations, where each unique combination of protein-ligand conformations is regarded as a replica of the system. Although replicas can be subjected to MMC simulations at different temperatures, only one temperature is currently used. For a given docking task, the corresponding ensembles of independent replicas are suitable for coarse-grained parallel computing. Moreover, a fine-grained parallelization takes place at the level of pairwise interactions between data points within each replica. These interactions are computed as three matrices, protein_ColumnVector_ × ligand_RowVector_ (*PRT*), KDE_ColumnVector_ × ligand_RowVector_ (*KDE*), and MCS_Matrix_ × ligand_ColumnVector_ (*MCS*). Here, a fairly large number of computations are subjected to fine-grained parallelization; the analysis of input data reveals up to 10^4^ data points for a single replica, which is sufficient to saturate computing resources available on modern CPUs and accelerators.

**Fig 3:**
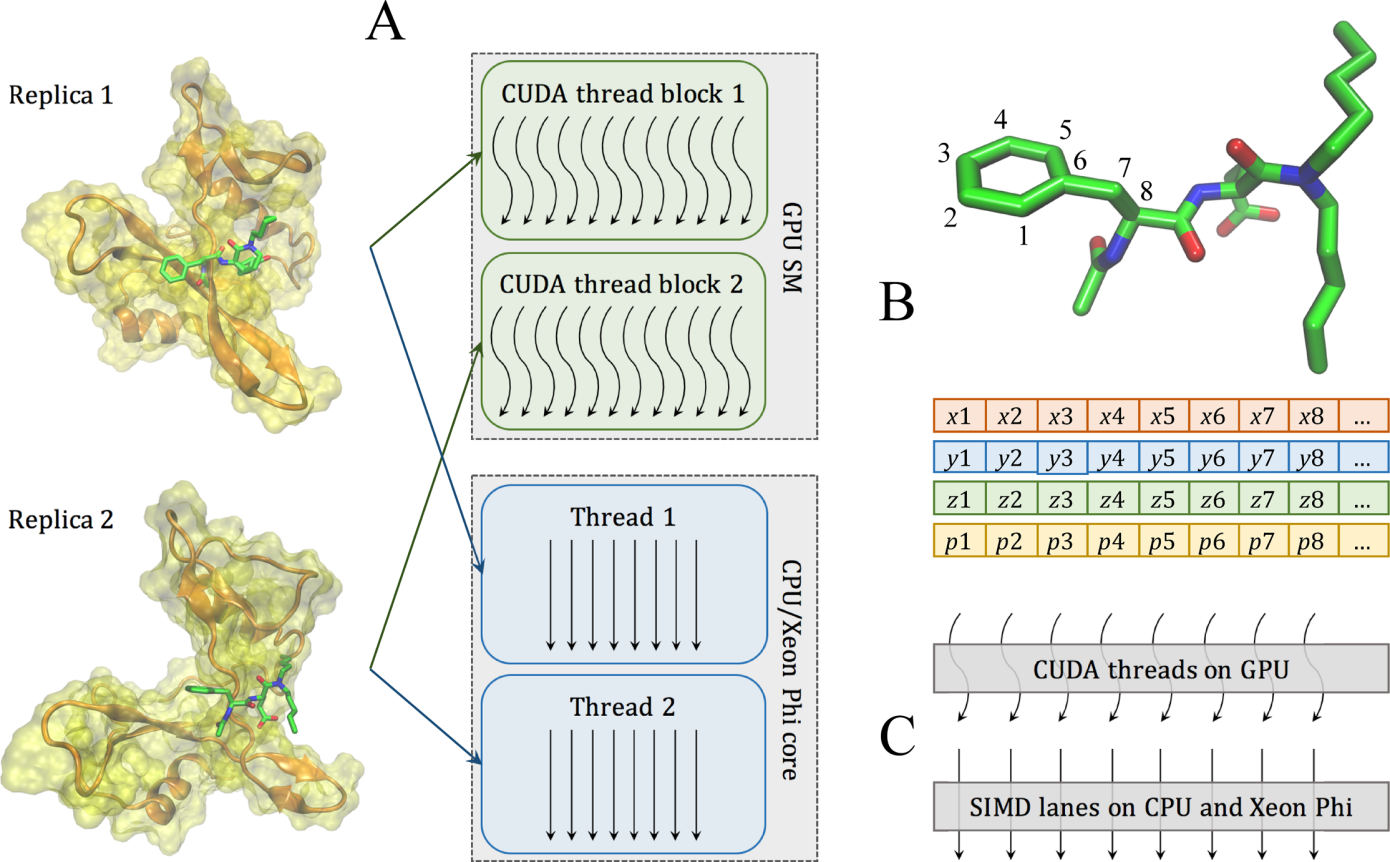
Two levels of parallelism in the docking kernel. (**A**) At the coarse-grained level, individual replicas are assigned to different CUDA thread blocks on GPU streaming multiprocessors (SMs) and different threads on CPU/Xeon Phi cores. (**B**) At the fine-gained level, data points for each replica are organized as Structure of Arrays containing Cartesian coordinates *x*, *y*, *z*, and parameters *p* associated with atoms, such as type, charge, and etc. Parameters for neighboring atoms are placed closely in memory to ensure the best execution efficiency. (**C**) Data points at the fine-gained level are accessed in parallel by CUDA threads on GPU and SIMD lanes on CPU and Xeon Phi.

Back-end calculations start when a task is fetched from the task pool. Fig 3 and Table 2 explain mapping between the docking algorithm and computing resources. First, replicas within each task are mapped to coarse-grained resources, GPU streaming multiprocessors (SMs) as well as CPU and Xeon Phi cores (Fig 3A and Table 2, Coarse-grained parallelism). When multiple GPUs are available, replicas within a given task are evenly assigned to the attached GPU cards. Second, interaction-level calculations (Fig 3B) are mapped to fine-grained resources, where computing 2D matrices utilizes SIMD lanes on CPU and Xeon Phi, and CUDA threads on GPU (Fig 3C and Table 2, Fine-grained parallelism). S1 Code illustrates loop operations on *PRT*, *KDE*, and *MCS* matrices involving a number of summation reductions. For instance, five energy terms calculated using the *PRT* matrix ($E_{ele}^{soft},\;E_{vdW}^{soft}$, *E*_*HB*_, *E*_*CP*_, and $E_{CP}^{PS}$) are directly reduced from a 2D array to a scalar value. Another type of reduction is hierarchical, where a 2D array *a*[*i*][*j*] is first reduced to a 1D array *b*[*i*] along the *j*-dimension, and then to a scalar value along the *i*-dimension. This technique is applied to selected data across all three matrices, e.g. *E*_*HP*_ in the *PRT* matrix, *E*_*KDE*_ in the *KDE* matrix, and *E*_*MCS*_ in the *MCS* matrix. In order to implement hierarchical reductions on GPU, we made adjacent GPU threads efficiently exchange data by scheduling the *i*-dimension as the outer loop, and the *j*-dimension as the inner loop. Specifically, the outer (inner) loop iterates over ligand_RowVector_ (protein_ColumnVector_) for the *PRT* matrix, ligand_RowVector_ (KDE_ColumnVector_) for the *KDE* matrix, and rows of MCS_Matrix_ (columns of MCS_Matrix_) for the *MCS* matrix.

**Table 2:** Algorithm mapping to hardware and software models of coarse- and fine-grained parallelism in GeauxDock.

Algorithm mapping	Platform	Hardware model	Software model
***Coarse-grained parallelism***			
11–550 replicas	CPU	4–10 cores with 2-way multi-threading	8–20 threads
	Xeon Phi	60 cores with 4-way multi-threading	240 threads
	GPU	16 streaming multiprocessors	CUDA thread blocks
***Fine-grained parallelism***			
~10,000 pairwise interactions	CPU	two 256-bit AVX SIMD instructions per cycle	8 SIMD lanes (SP)
	Xeon Phi	one 512-bit SIMD instruction per cycle	16 SIMD lanes (SP)
	GPU	192 scalar processors with multi-threading	CUDA threads

AVX–Advanced Vector Extensions; SIMD–single instruction, multiple data; SP–single-precision calculations.

**Fig 4:**
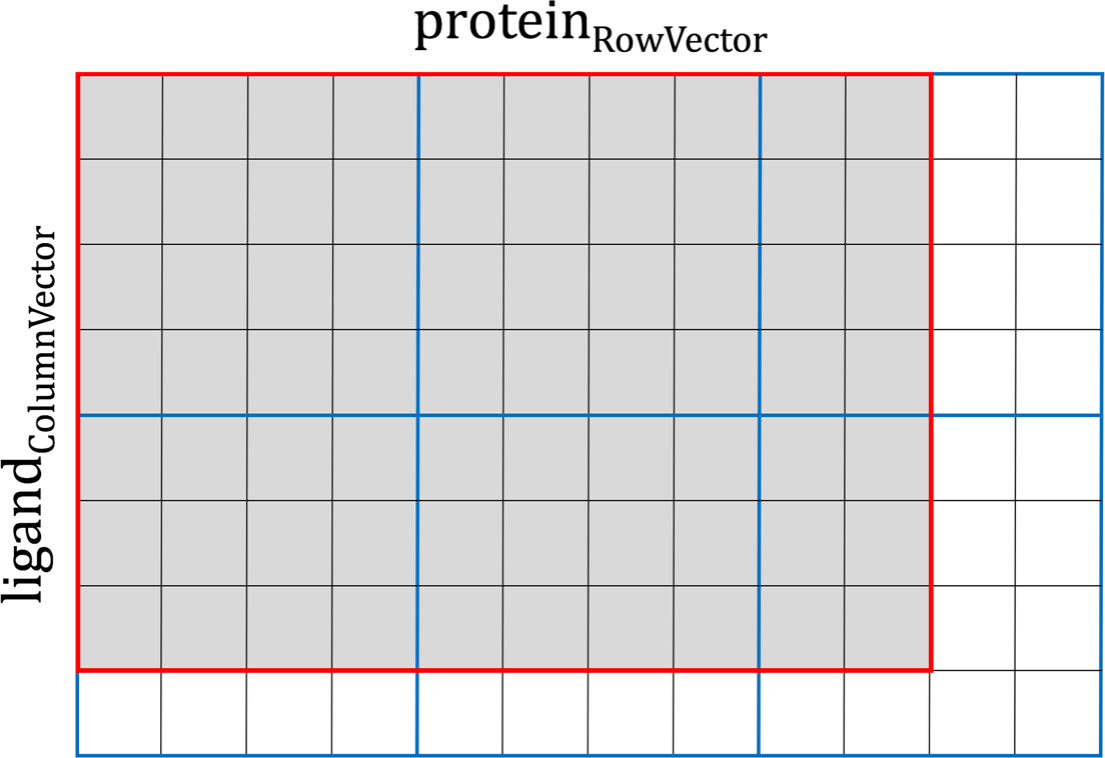
Example of parallel calculations for a data matrix. A small, 96-element matrix ligand_ColumnVector_ × protein_RowVector_ is outlined in red, whereas the 4 × 4 CUDA thread block iterating over the matrix is outlined in blue. Here, at least 6 cycles are required to process the data matrix utilizing a total of 70 parallel threads (gray cells), while the remaining 26 threads are idle (white cells). An optimal shape of CUDA thread blocks can be constructed dynamically to improve the computational performance by reducing the number of cycles required to traverse the data matrix.

2D CUDA thread blocks are responsible for calculations on GPU (Fig 3A, green rounded boxes). The shape and size of CUDA thread blocks are flexible and can be tuned for the optimal performance. Given that the CUDA warp size is fixed at 32, the *x*-dimension of the CUDA thread block is best defined as a multiple of 32. Also, the maximum number of 1,024 threads per CUDA thread block restricts the *y*-dimension, for example, the size of the *y*-dimension cannot be greater than 32 when *x*-dimension is 32, because 32 × 32 = 1024. However, the shapes of 2D interaction matrices do not always perfectly match those of CUDA thread blocks. For instance, the *x*-dimension is always greater than the *y*-dimension in *PRT* and *KDE* matrices, whereas a typical *MCS* matrix has the *y*-dimension greater than the *x*-dimension. Therefore, boundary conditions require a careful design of CUDA thread blocks to leave a certain number of idle threads for the thread management. This procedure is illustrated in Fig 4, where processing a small, 70-element data matrix (outlined in red) requires at least six cycles of a 4 × 4 CUDA thread block (each cycle is outlined in blue). With this setup, 70 parallel threads are fully utilized (gray cells), leaving 26 threads idle (white cells). Overall, the number of CUDA threads is fixed at the compiling time, but the optimal shape of the thread block is defined at the runtime, when the input data become available. Here, the objective is to find the best combination of *x*- and *y*-dimensions consuming the least amount of computing cycles to traverse the data matrix, where a computing cycle is defined as follows:

cycle=(ceiling(data_size_x/cuda_threads_x))×(ceiling(data_size_y/cuda_threads_y)) Eq. 1

In practice, only a handful of configurations are valid; we enumerate and evaluate these configurations to find the optimal solution. As an example, using Tesla K20Xm GPU with 1,024 threads per thread block, a typical configuration for *PRT*, *KDE*, *MCS* matrices is 128 × 8, 128 × 8, and 32 × 32, respectively.

Different from the GPU version, the back-end for CPU implemented in C++ with OpenMP pragmas assigns processor threads to carry out computations for individual replicas (Fig 3A, blue rounded boxes). In order to avoid thread migration and ensure the best cache locality, the environment variable "OMP_PROC_BIND" is set to "true". In addition, inner loops in data computations iterating over protein_ColumnVector_ (*PRT* matrix), KDE_ColumnVector_ (*KDE* matrix), and columns of MCS_Matrix_ (*MCS* matrix) are marked with vector pragmas to assist Intel compiler in generating an efficient, vectorized code. Note that the same CPU code can be used on Xeon Phi since almost all performance tuning techniques for CPU apply to this accelerator as well. The major difference is that the code for Xeon Phi is required to be offloaded to the accelerator, which is conceptually similar to GPU programming. The offload is accomplished using compiler pragmas, i.e. “#pragma offload target (mic) in (data_in) out (data_out)”. However, the present pragma-based Xeon Phi programing model was designed to offload a block of code to only one device. The current implementation of GeauxDock works only with a single Xeon Phi card. Although replicas could be distributed manually across multiple accelerators, one should keep in mind that at least 240 replicas are required to effectively utilize Xeon Phi. Since docking tasks have no more than 550 replicas, splitting the workload among multiple Xeon Phi cards would inadvertently decrease the overall performance. In addition, any code modification targeting the Xeon Phi platform would complicate the code maintenance. In fact, workload sharing at the task level represents a more practical and scalable approach, which will be implemented in the future release of GeauxDock.

### Data structure

A docking task contains complex data, including read-only protein and ligand conformations, MMC simulation parameters, MCS, KDE and PSP force field parameters, as well as the dynamic configuration and output data from individual replicas. GeauxDock employs the Structure of Arrays (SoA) to store the data ensuring the best data locality. For example, the SoA for the ligand conformation shown as S2 Code A contains elements *x*[*L*], *y*[*L*], *z*[*L*], *t*[*L*], and *c*[*L*], representing *x*, *y*, *z* coordinates, the type, and electric charge for all ligand atoms, respectively. *L* defines the maximum number of ligand atoms and it is set at the compiling time. Fig 3B shows that the data associated with neighboring atoms are stored in consecutive memory addresses in order to maximize the efficiency of memory operations required for the fine-grained parallelization. With this design, CUDA threads on GPU and SIMD lanes on CPU and Xeon Phi access these data in a stride-1 pattern as illustrated in Fig 3C. Data structures for protein conformations, MMC simulation parameters, and PSP, KDE and MCS force field parameters are created in a similar fashion. These data constitute the first-level SoA providing read-only information, and are used as building blocks to construct the multiple-replica simulation context.

**Fig 5:**
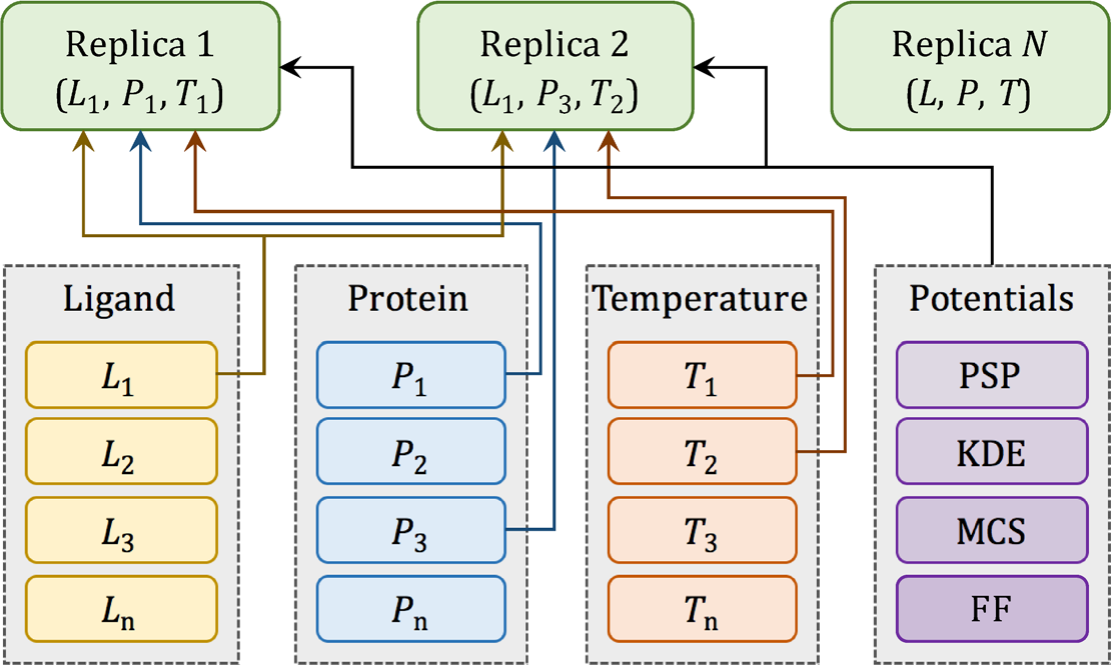
Data indexing for multi-replica Monte Carlo simulations. Individual replicas are multi-dimensional objects comprising different combinations of ligand (*L*) and protein (*P*) conformations, and temperatures (*T*), as well as the same set of PSP, KDE, MCS potentials and force field (FF) parameters. All these data are read-only, labeled with tags, and accessible through indexes as depicted by arrows.

To systematically assemble replicas from these raw data, we created a data structure called "ReplicaInfo", whose purpose is to assemble a replica from the raw data using indirect references to various arrays. The concept of ReplicaInfo is presented in Fig 5, where two example replicas, (*L*_1_, *P*_1_, *T*_1_) and (*L*_1_, *P*_3_, *T*_2_), are created using indexes to the same ligand conformation (*L*_1_), but different protein conformations (*P*_1_ and *P*_3_) and simulation temperatures (*T*_1_ and *T*_2_). ReplicaInfo was designed to yield a high computational efficiency of data exchange between replicas during parallel tempering MMC simulations [30], which requires swapping only a few indexes rather than the associated large data arrays. Further, the ReplicaInfo structure is used to store the temporary simulation status, including energy values and ligand orientations with respect to the target protein pocket. Simulation logs are saved in the “Simlog” data structure, whose entry can also be found in ReplicaInfo. We note that the ReplicaInfo can be modified during MMC simulations, while the associated data are read-only.

In addition to the first-level SoA, we designed the second-level SoA called the “Complex” (S2 Code B) providing the outermost container for the computation data. The elements of Complex are various data structures, including protein and ligand conformations, MMC simulation parameters, MCS, KDE and PSP force field parameters, ReplicaInfo, and the data size. Essentially, a single instance of Complex SoA and Simlog hold all data associated with a computation task. Because the memory for Complex and Simlog is allocated only once, when either the CPU/Xeon Phi or GPU version of GeauxDock is initiated, it must be large enough to hold data for any docking tasks from the task pool. Docking calculations for the CCDC/Astex dataset require about 5 MB of memory for each Complex, whereas the entire Simlog would allocate about 1.5 GB of memory. In practice, only about 100 MB of Simlog data need to be transferred to the host and saved on disk.

### Data rearrangement

Irregular code patterns caused by dynamic data may significantly affect the performance. The docking kernel code contains conditional branches and indirect memory references, for example, calculating a branch path depends on the distance between a ligand atom and a protein point, which is changing in the course of MMC simulations. Although it is difficult to speed up the code containing these dependencies, we improved the code regularity for certain cases. For instance, incrementally sorting KDE data elements by the atomic type *t* helps improve the regularity of the conditional code “if (lig->*t*[index] = = kde->*t*[index])” in a loop iterating over hundreds of KDE data points. Another example is the indirect memory reference, such as “*d* = array[ligand->*t*[index]][protein->*t*[index]]”. Here, sorting ligand and protein objects by *t* greatly improves the locality of accessing array elements. Altogether, data rearrangement enhances the performance of GeauxDock by 9.6%, 12.2% and 8.2%, on CPU, Xeon Phi and GPU, respectively.

### Strength reduction

In order to further speed up calculations within the docking scoring function, the strength reduction technique is applied to reduce its computation complexity. Original mathematical formulas for various energy terms in the MMC kernel are divided into pre-processing and computation groups. The pre-processing combined with data transformation is conducted within the front-end of GeauxDock. An example is shown as S3 Code, where the indirect memory reference prtconf.r[index] is removed from the original kernel (S3 Code A) and included in the pre-processing stage (S3 Code B), leading not only to a better memory locality, but also to fewer instructions in the optimized kernel. Another technique used to accelerate computations within the docking kernel is the reduction of the arithmetic intensity. For instance, S4 Code A shows a part of the original kernel computing the soft van der Waals potential, which includes 6 loads, 9 multiplications, 3 division and 5 power functions. To speed up the MMC kernel, some calculations are either moved to the pre-processing step or executed between certain blocks of the code and then reused when calculating the potential. As the result, the optimized code shown as S4 Code B has only 2 loads, 6 multiplications, 3 divisions and no power functions.

### Architecture specific optimization

The power of accelerators can be fully utilized only when time is primarily spent on computations rather than data communication. GeauxDock is implemented based on this principle by moving compute-intensive MMC simulations to Xeon Phi and GPU. S5 Code shows the MMC conformational sampling in ligand docking. First, a new configuration of a ligand is generated by randomly perturbing the present configuration. Next, the energy of the new configuration is calculated and compared to the energy of the old configuration using the Metropolis algorithm [31]; the new configuration is accepted with a certain probability to be used in the next iteration, otherwise it is rejected. Even though some components of the docking kernel, such as evaluating the Metropolis criterion, are less suitable for the parallelization on GPU and Xeon Phi, this approach yields a better overall performance than offloading parts of the docking kernel. For instance, offloading only energy calculations could potentially generate an excessive communication between the host and the accelerator. In that case, advanced optimization techniques such as the asynchronous kernel execution and data copying between multiple tasks would have to be applied for a better performance. However, because extra communication is avoided in the MMC kernel, the code requires no further optimization of data transfer.

For GPU, the memory is carefully managed within the GeauxDock code with heavily reused variables, such as interaction distances, placed in registers. Moreover, the shared memory is used for those frequently reused data, such as ligand coordinates and energy parameters, which may have an irregular access pattern. Large arrays with the stride-1 parallel access pattern are defined as SoA, sorted for improved regularity, and saved in the global memory. Importantly, level 1 data cache on Tesla K20Xm GPU does not buffer the global memory traffic by default. The docking kernel has a good reuse pattern for *PRT* and *KDE* matrices, therefore, inserting _ldg intrinsic enables the level 1 data cache mechanisms to enhance memory operations. This technique improves the GPU performance by 4% for *PRT* and *KDE* matrices. In contrast, the cache optimization cannot be applied to computations for the *MCS* matrix, which have no global data reuse at all.

Since the docking kernel invokes reduction operations, partial results in each CUDA thread need to be added to a scalar value. Here, a simple implementation stores temporary data in the shared memory, where the amount of the required memory scales linearly with the number of CUDA threads. In the early version of GeauxDock, the capacity of the shared memory limited the maximum number of CUDA threads per thread block to 768. Since using more CUDA threads per block generally delivers a better performance on Tesla K20Xm GPU, the current docking kernel uses __shfl and __shfl_xor intrinsic instructions for reduction operations. This technique enables a direct data exchange between CUDA threads without consuming the shared memory. Not only is the new reduction code 3× faster, but it also allows to use 1,024 CUDA threads per block improving the overall performance by 40%. Finally, many elementary functions, exp, log, sin, cos, etc., are frequently used in the docking kernel. The CUDA math library offers accelerated versions of these math functions [19], which are enabled by the “-use_fast_math” compiler flag. This tuning yields a 30% performance boost, however, the fast math intrinsic for GPU is not guaranteed to be fully compatible with the IEEE floating point standard. Nonetheless, a careful comparison of the results against the CPU code shows that the error rate is smaller than 0.0001%.

### Performance evaluation

The performance of MMC kernels in GeauxDock is evaluated on several computing platforms using diverse input data. We conducted benchmarking calculations using four Linux computers listed in Table 3, including a mainstream PC desktop, a PC desktop with the latest consumer grade GPU, a heterogeneous HPC cluster node with both GPU and Xeon Phi accelerators, and an HPC cluster node with two GPU cards. We set the optimization level to “-O3” with the following additional flags for the Intel compiler: “-fno-fnalias -ansi-alias -fargument-noalias” (to safely remove pointer aliases), “-ipo” (to enable interprocedural optimization), “-vec-threshold0” (to enable vectorization whenever possible), and “-fma” (to enable the fused-multiplication-add code generation). Architectural events listed in Table 4 were recorded by hardware counters using the Performance Application Programming Interface (PAPI) library version 5.4.0 [32]. In addition, we implemented timers directly in the code in order to measure the execution time of an arbitrary segment of the code. We noticed that time measurements have minor fluctuations of ~5%, therefore, all timings are reported as the average over 8 independent runs.

**Table 3:** Hardware and software specification of four computing platforms used to evaluate the performance of GeauxDock.

Platform	Processor	Accelerator	Compiler
D1 (desktop)	1 × Intel Core i7-2600, 4c, 8t, 3.4GHz, Turbo	-	Intel 14.0.2
D2 (desktop)	1 × Intel Xeon E5-2620, 6c, 12t, 2.0GHz, Turbo	1 × GeForce GTX 980	GCC 4.4.7, CUDA 7.0
C1 (HPC cluster)	2 × Intel Xeon E5-2680 v2, 10c, 10t, 2.8GHz, Turbo	1 × Tesla K20Xm, 1 × Intel Xeon Phi 7120P	Intel 14.0.2, CUDA 6.5
C2 (HPC cluster)	2 × Intel Xeon E5-2670, 8c, 8t, 2.6GHz, Turbo	2 × Tesla K20Xm	Intel 14.0.2, CUDA 5.5

HPC–high-performance computing; c–the number of cores; t–the number of threads; Turbo–a dynamic frequency scaling to modify the CPU clock rate based on the number of active cores and thermal conditions.

**Table 4:** PAPI preset events used to assess the code performance.

PAPI event	Description
PAPI_LI_DCM	Number of level 1 data cache misses
PAPI_BR_MSP	Number of branch mispredictions
PAPI_TOT_INS	Total number of instructions
PAPI_TOT_CYCLES	Total number of CPU cycles

### Benchmarking dataset

Benchmarking calculations are carried out for a single target protein, the pp60(c-src) SH2 domain complexed with ace-malonyl Tyr-Glu-(N,N-dipentyl amine) (PDB-ID: 1a07) [33] and a set of 204 drug compounds selected from the CCDC/Astex dataset [34]. 1a07 represents a typical docking target with 344 protein effective points and an ensemble of 11 protein conformations. Depending on the number of rotatable bonds, up to 50 conformations are generated for ligands, thus the ensemble-based docking employs up to 550 replicas (11 × 50) of individual systems. In addition to this default protocol, we test the code scalability using a varying number of replicas at multiple temperatures. Other parameters affecting the computational complexity are the number of non-hydrogen ligand atoms and the number of points to compute the evolution-based components of the GeauxDock force field, KDE and MCS. Although both KDE and MCS scoring terms are used to calculate various restraints derived from homology rather than physical interactions, these points are iterable from the computing point of view. Therefore, KDE and MCS interacting points are equivalent to ligand atoms and protein effective points in the physics-based components of the GeauxDock force field.

## Results and Discussion

### Dataset and simulation characteristics

**Fig 6:**
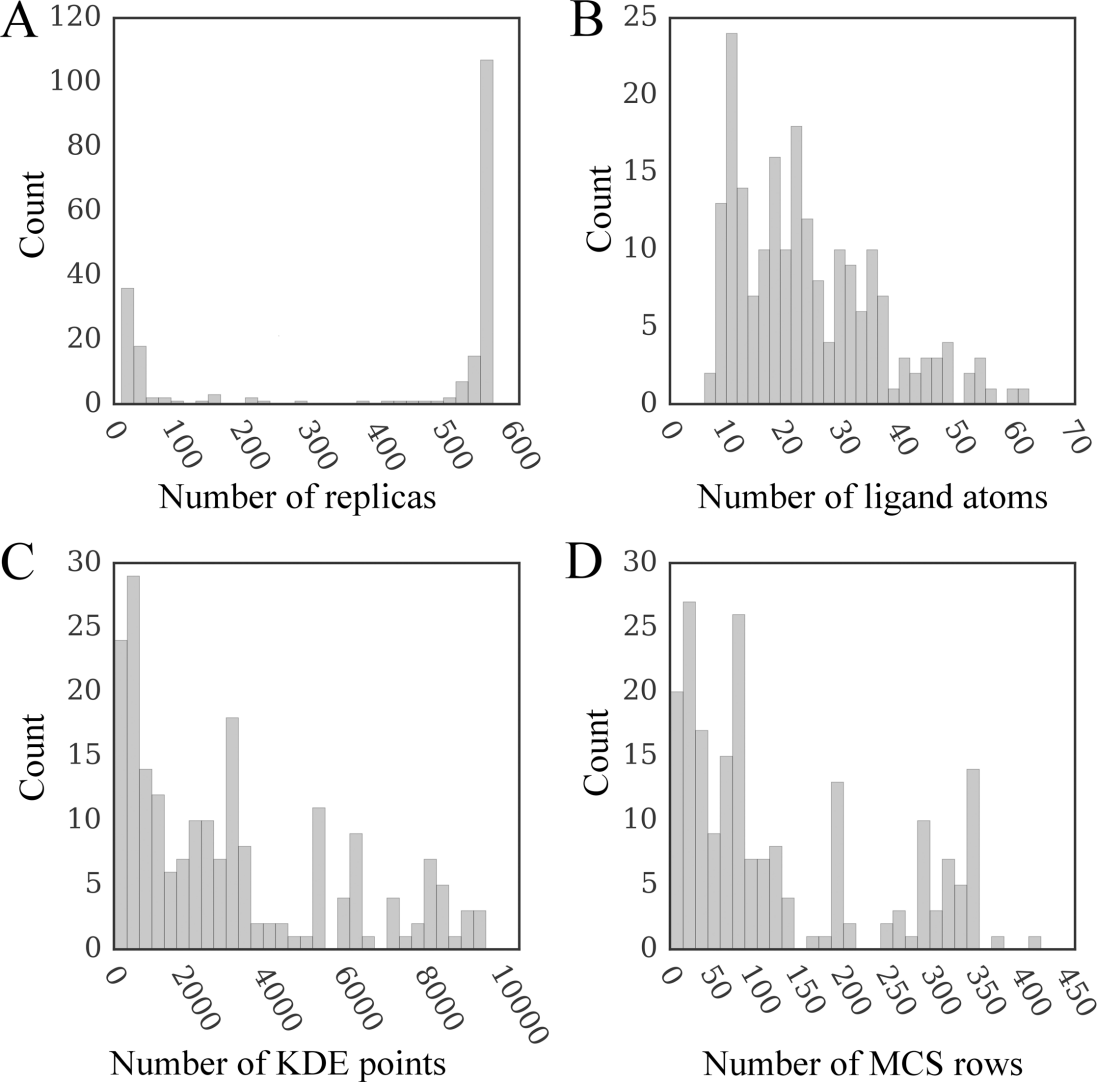
Distribution of various parameters affecting docking time. The number of (**A**) replicas, (**B**) ligand non-hydrogen atoms, (**C**) KDE points, and (**D**) rows in the MCS matrix are shown for the dataset of 204 CCDC/Astex compounds. KDE (Kernel Density Estimation) and MCS (Maximum Common Substructure) points are used to calculate evolution-based components of the docking force field.

The distributions of the number of replicas, ligand atoms, as well as KDE and MCS points are shown in Fig 6. GeauxDock employs multiple replicas to account for the flexibility of protein-ligand complexes, where each replica contains a unique combination of protein and ligand conformations. The highest peak in Fig 6A at around 550 replicas corresponds to highly flexible compounds with multiple rotatable bonds, whereas the smaller peak at around 11 replicas represents those rigid complexes having only a single conformer. Given that the hydrogen atoms are omitted when counting atoms, the range between 6 and 62 heavy atoms presented in Fig 6B agrees well with the qualifying range for drug molecules according to the extended version of Lipinski’s rule-of-five [35]. Because KDE points and rows in MCS_Matrix_ are calculated using template-bound ligands detected by the *e*FindSite algorithm [28,36] their distributions (Fig 6C and 6D, respectively) depend on the number and size of ligands extracted from holo-templates.

Another important simulation parameter is the number of MMC cycles. We found that 1,000 MMC cycles is sufficient for production runs to converge. Since these calculations require 4.8 to 61 minutes on various platforms, the average wall time for the docking kernel is 1.4 seconds on the fastest machine (platform D2, Table 3) and 18 seconds on the slowest computer (platform D1, Table 3). Because the number of replicas (up to 550) is multiplied by the number of temperatures (up to 240) in our benchmarks, and several versions of the docking code needed to be tested, the time required to complete simulations could be hundreds times longer than that for production runs. Therefore, shorter simulations with 100 MMC cycles are used for benchmarking purposes.

### Performance of docking kernel with an ample coarse-grained parallelism

The execution time for docking kernels includes not only computations but also time required for the data transfer to and from accelerator devices. Moreover, the kernel performance can be affected by the ensemble size (the number of replicas), because those docking systems containing rigid ligands provide insufficient coarse-grained parallelism to fully utilize computing resources. On that account, we first need to determine the ideal performance as well as a performance penalty caused by the meager coarse-grained parallelism. To address this problem, we conducted a series of simulations providing a sufficient number of replicas to deliver an ample coarse-grained parallelism. Specifically, we used 400 replicas for a dual CPU with 20 cores and 20 threads, 2,400 replicas for Xeon Phi with 60 cores and 240 threads, and 280 replicas for GPU with 14 streaming multiprocessors and 14 CUDA thread blocks.

**Fig 7:**
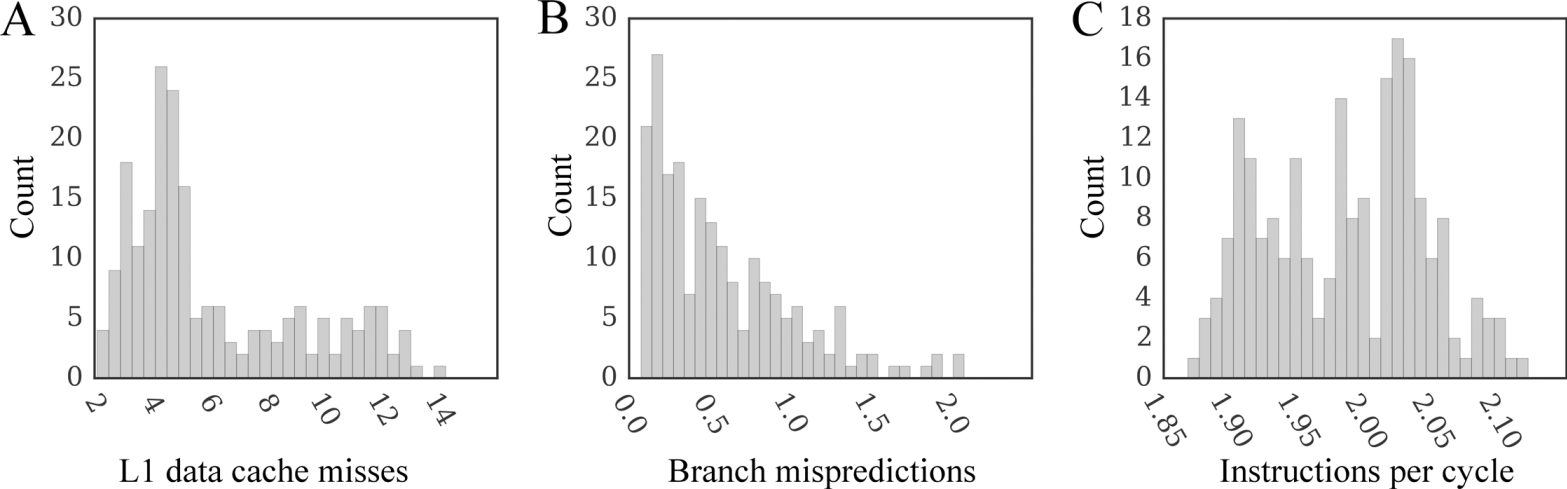
Performance characteristics for a single-threaded docking kernel on CPU. The number of (**A**) level 1 data cache misses per 10^3^ instructions, (**B**) branch mispredictions per 10^3^ instructions, and (**C**) instructions per cycle.

**Fig 8:**
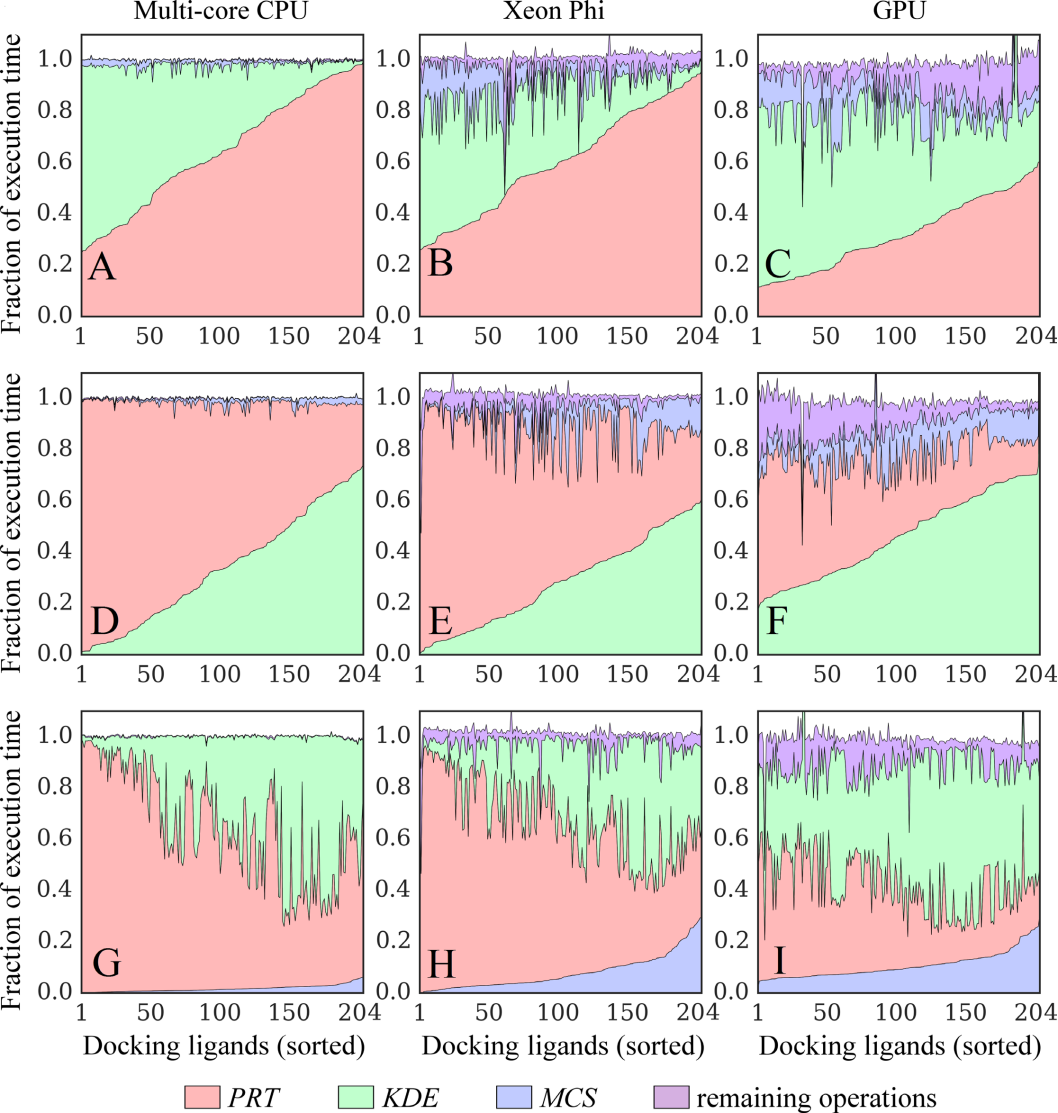
Time breakdowns for docking kernels running on different platforms. Kernel implementations for (**A**, **D**, **G**) multi-core CPU, (**B**, **E**, **H**) Xeon Phi, and (**C**, **F**, **I**) GPU are tested. Three major operations compute the following interaction matrices: protein_ColumnVector_ × ligand_RowVector_ (*PRT*, green), KDE_ColumnVector_ × ligand_RowVector_ (*KDE*, red), and MCS_Matrix_ × ligand_ColumnVector_ (*MCS*, blue). Purple areas correspond to the remaining operations. KDE (Kernel Density Estimation) and MCS (Maximum Common Substructure) points are used to calculate evolution-based components of the docking force field, whereas the *PRT* matrix is used to calculate the majority of physics-based potentials. Results collected for the dataset of 204 CCDC/Astex compounds are sorted on the *x*-axis with respect to increasing time of computing (**A**, **B**, **C**) *PRT*, (**D**, **E**, **F**) *KDE*, and (**G**, **H**, **I**) *MCS* matrices.

The performance of docking kernels on CPU is assessed using the C1 computing system (Table 3). We first evaluate the serial performance by enabling only 1 thread on a single processor core. Using the total number of CPU cycles according to the PAPI event PAPI_TOT_CYCLES (Table 4) and the computing time measured by either the PAPI timer or our timer, the average dynamic CPU clock rate is 3.58 GHz ±0.02. Fig 7 shows several characteristics assessing the overall computational performance of the docking code. Computing *PRT* and *KDE* matrices are the major components of the docking kernel (Fig 8A and 8D). Since the maximum reuse distances [37] for these data (300 and 9000, respectively) are small enough to fit L1 data cache, the cache efficiency in GeauxDock is very high. Indeed, in most cases, the number of L1 data cache misses per 10^3^ instructions is less than 7 (Fig 7A), which is lower compared to a broad distribution of 5–30 misses reported for thoroughly tuned SPEC CPU2006 benchmark kernels [38] tested on the same CPU microarchitecture. Applying an additional loop tiling transformation [37] to further reduce the reuse distance does not improve the performance. Similarly, the number of branch mispredictions per 10^3^ instructions for the SPEC CPU2006 kernels is between 1 and 10 [38], therefore, the docking code is superior with no more than 2 branch mispredictions (Fig 7B). Moreover, GeauxDock achieves an average instruction throughput rate of about 2, which is notably higher than 1.43 instructions per cycle reported for the most efficient SPEC CPU2006 kernel [38]. This comparison with the SPEC CPU2006 benchmark suite demonstrates that the serial, CPU version of the docking kernel in GeauxDock is indeed highly optimized.

**Fig 9:**
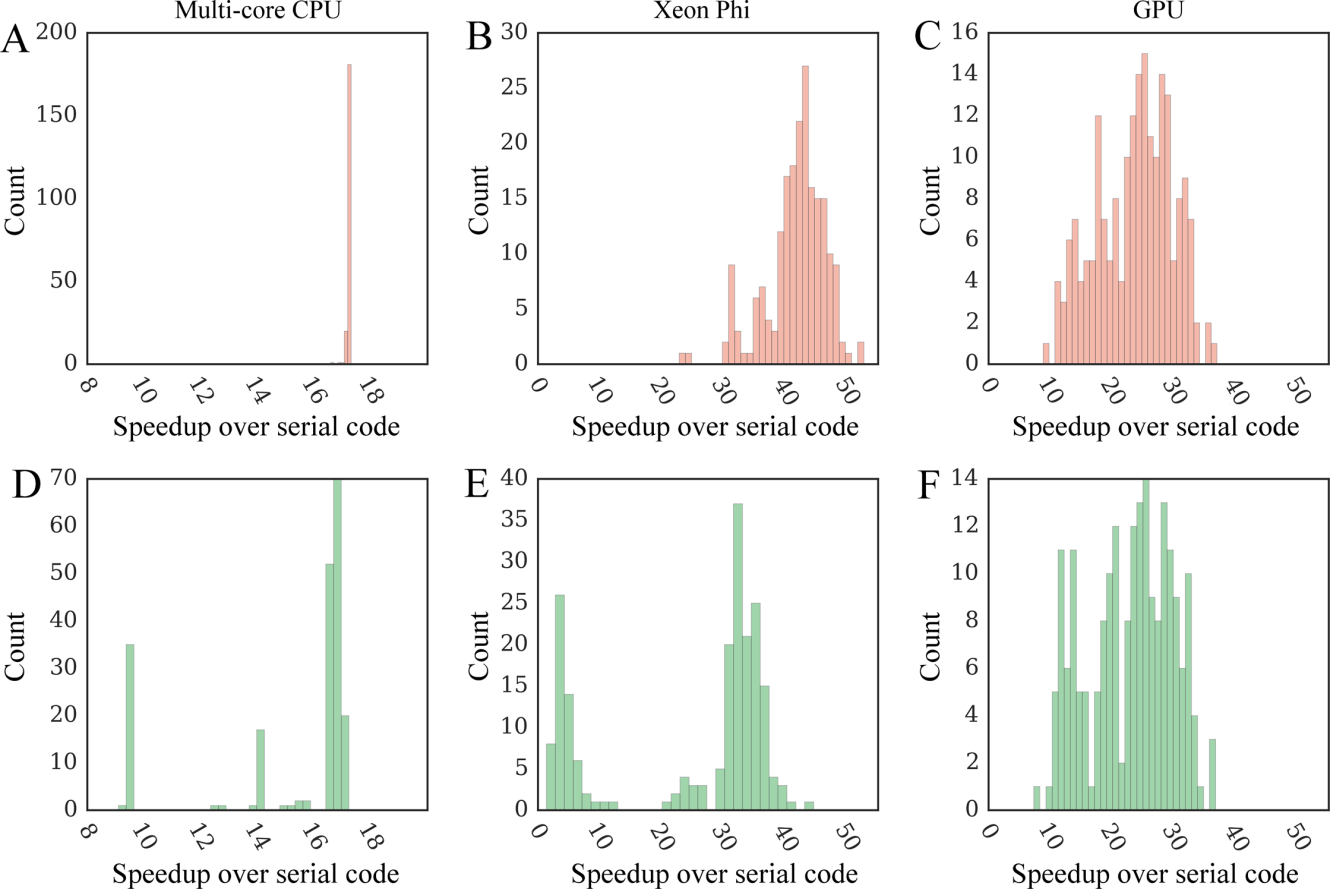
Distribution of speedups of parallel GeauxDock over the serial CPU version. Benchmarking calculations are conducted for the dataset of 204 CCDC/Astex compounds using (**A**-**C**, red) modified input data providing an ample coarse-grained parallelism and (**D**-**F**, green) unmodified input data. Three kernel implementations are tested for (**A**, **D**) multi-core CPU, (**B**, **E**) Xeon Phi, and (**C**, **F**) GPU.

**Fig 10:**
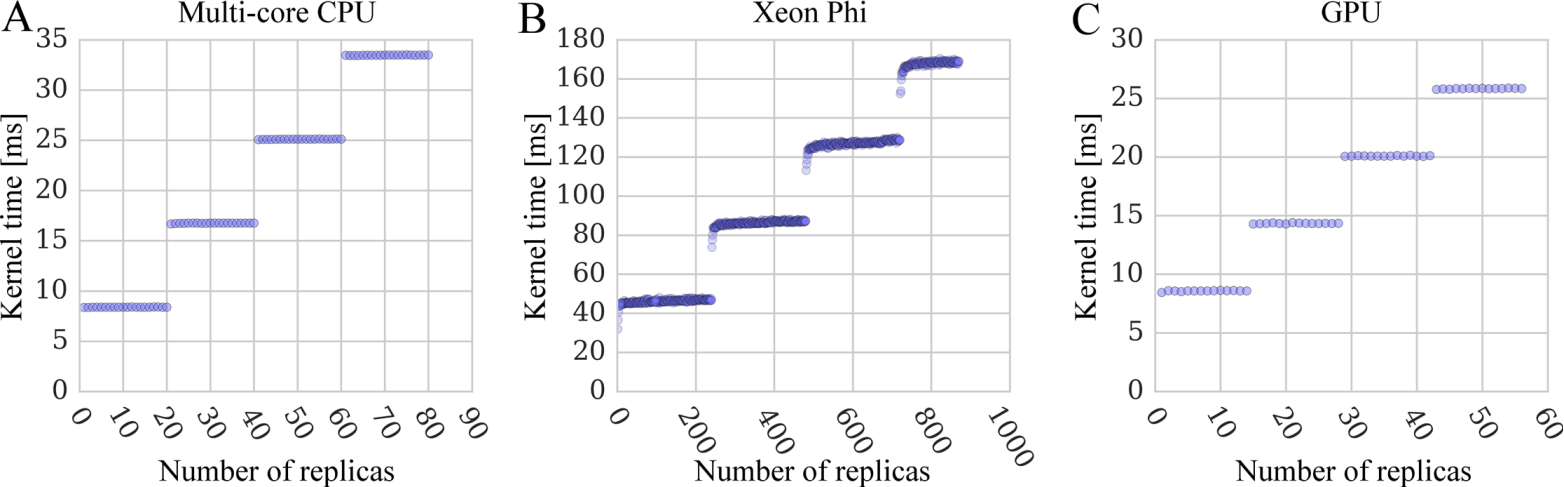
Performance scaling of docking kernels with different numbers of system replicas. Benchmarking calculations are performed using (**A**) multi-core CPU, (**B**) Xeon Phi, and (**C**) GPU. The width of horizontal lines is 20 replicas for a dual 10-core CPU, 240 for a 60-core Xeon Phi with 4-way multi-threading, and 14 for a 14-multiprocessor GPU.

Next, using the optimized serial CPU code as a baseline, we measure the performance of the parallel versions of GeauxDock on a dual multi-core CPU, Xeon Phi and GPU using the C1 computing system (Table 3). Enabling 20 threads on a dual CPU triggers the dynamic frequency scaling and decreases the average CPU clock rate to 3.07 GHz ±0.11. Fig 9A shows that the average speedup of multi-threaded GeauxDock over its serial version is 17.22× ±0.06, which actually corresponds to the maximum theoretical speedup accounting for the lower clock rate (20 × 3.07 GHz / 3.58 GHz). Compared to the serial code, the parallel docking kernel runs from 22× to 56× faster on Xeon Phi 7120P (Fig 9B) and 10× to 38× faster on Tesla K20Xm GPU (Fig 9C). One should bear in mind that the simulation time depends on not only the data size, but also the relative amount of *PRT*, *KDE* and *MCS* computations. Further, the irregular portions of the docking code are handled differently by various devices because of their architectural characteristics causing variations across the dataset. As we mentioned in the introduction section when discussing hardware design, the simpler computing units of Xeon Phi and GPU are more susceptible to dynamic branches than sophisticated CPU cores.

### Performance of docking kernel on real data

Next, we test the parallel performance of each platform against realistic workloads. Figs 9D and 8F show that multi-threaded CPU and GPU versions of the docking kernel generally maintain their high performance on real data. In contrast, the performance of Xeon Phi is significantly affected by the lack of an ample coarse-grained parallelism (Fig 9E). Although the co-processor is twice as fast as a dual CPU in 71.1% of the cases (a speedup of 17× and more), Xeon Phi performs about twice as slow as a dual CPU for the remaining docking systems. This double peak pattern matches the bimodal distribution of the number of replicas shown in Fig 6A, demonstrating that the computational throughput of Xeon Phi is significantly affected by those workloads providing insufficient coarse-grained parallelism.

To further investigate the effect of the number of replicas on the parallel performance, we compiled a separate testing dataset comprising a single conformation of the target protein 1a07 and a rigid ligand adamantanone (PDB-ID: 5cpp) [39]. This docking system is replicated *n* times at different temperatures to strictly control the number of replicas in docking simulations. The docking time for multi-core CPU, Xeon Phi and GPU kernels are presented in Fig 10. Fig 10A and 10C show sets of horizontally parallel lines with even vertical distances, whose width corresponds to the number of CPU cores and GPU streaming multiprocessors, respectively. Here, replicas are processed in parallel by independent computing units with the execution time equal to the number of replicas divided by the core count. The width of horizontal lines for Xeon Phi shown in Fig 10B is 240 because of the hardware multi-threading (60 cores × 4 threads per core). Clearly, it is beneficial to place 4 threads on a single core in order to fully utilize the hardware. Moreover, the kernel time for the first few data points at the beginning of each horizontal line is somewhat shorter demonstrating that the co-processor performance is affected by the global resource contention.

### A reliable model for the docking performance

To further understand the performance characteristics, we analyze various components of the docking kernel including the time spent on computing *PRT*, *KDE*, and *MCS* interaction matrices. *KDE* and *MCS* data are used to calculate evolution-based components of the docking force field, whereas the *PRT* matrix is used to calculate physics-based potentials. The time spent on computing the remaining operations is measured using a modified kernel, in which *PRT*, *KDE*, *and MCS* calculations are disabled. Fig 8 shows time contributions from these four components. Computing *PRT* contributes to 64.4%, 60.4%, and 32.1% of the total execution time on CPU, Xeon Phi, and GPU, respectively (Fig 8A–8C). The percentage of the kernel time for *KDE* is 33.9% on CPU, 28.2% on Xeon Phi, and 46.3% on GPU (Fig 8D–8F), whereas for *MCS*, it is 2.7% on CPU, 5.1% on Xeon Phi, and 10.4% on GPU (Fig 8G–8I). The remaining operations make up about 10% of the total kernel time on Xeon Phi and GPU. In contrast, these computations require almost no time on CPU because the sophisticated processor cores handle sequential workloads (e.g. updating ligand coordinates, generating random numbers, calculating Metropolis acceptance criterion, etc.) as efficiently as highly parallel workloads. Further, the CPU code has no data transfer between the host and the accelerator, which is required only for Xeon Phi and GPU.

**Fig 11:**
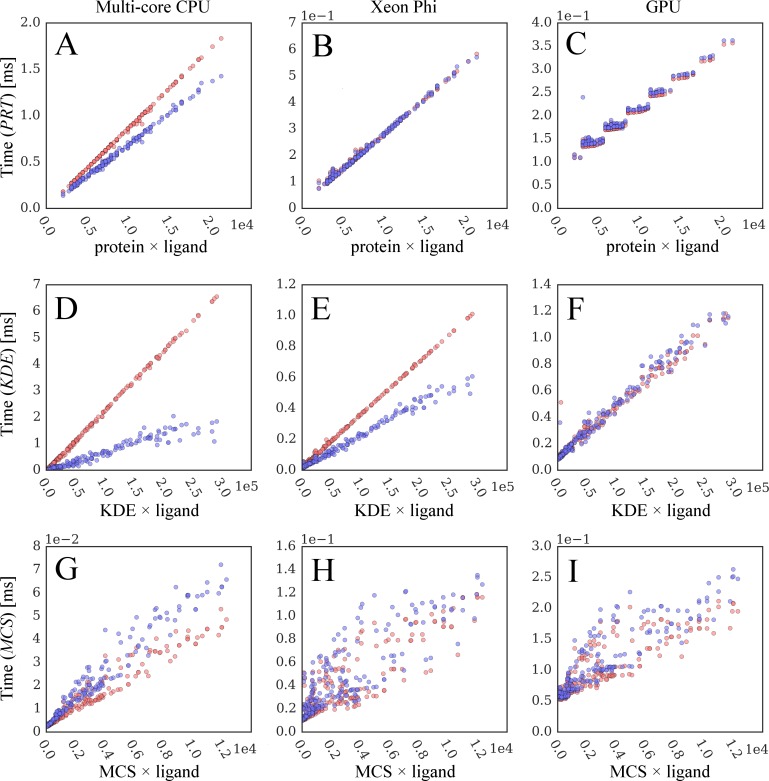
Correlation between computing time and static data size. Blue points are collected from original GeauxDock, whereas red points correspond to a modified docking code, where dynamic branches are turned off forcing the execution of all instructions. Three major operations compute (**A**-**C**) protein_ColumnVector_ × ligand_RowVector_ (*PRT*), (**D**-**F**) KDE_ColumnVector_ × ligand_RowVector_ (*KDE*), and (**G**-**I**) MCS_Matrix_ × ligand_ColumnVector_ (*MCS*) matrices. Three kernel implementations are tested for (**A**, **D**, **G**) multi-core CPU, (**B**, **E**, **H**) Xeon Phi, and (**C**, **F**, **I**) GPU.

Next, we analyze the correlation between the computing time and the static data size. In addition to the original docking code, we examine the performance impact of dynamic branches by forcing the calculation of all operations; this modified implementation is referred to as a "regulated" code. Fig 11 shows the correlation between the execution time and the data size for the original program in blue and the regulated code in red. Fig 11A–11F demonstrate that the time required to calculate the *PRT* (*KDE*) matrix strongly correlates with its size; the coefficient of determination, *R*^2^, for the original code shown in blue is 0.996 (0.938) for CPU, 0.996 (0.987) for Xeon Phi, and 0.952 (0.981) for GPU. This correlation is somewhat weaker for the *MCS* matrix with the *R*^2^ of 0.957, 0.720 and 0.793 for CPU, Xeon Phi and GPU, respectively. Forcing the execution of the entire code by eliminating dynamic branches has two major effects on the kernel performance. First, it improves the correlation between the computing time and the data size, for instance, the *R*^2^ for the *KDE* matrix shown in red in Fig 11D–11F is 0.999 for CPU and Xeon Phi, and 0.983 for GPU. Second, the regulated code is slower, however, the relative increase of the execution time is clearly architecture-dependent. In general, CPU skips executing most of the instructions downstream of branches because their conditional outcome can be accurately predicted, which yields a better performance (Fig 11A and 11D). The performance of GPU (Fig 11C and 11F) is unaffected by branches indicating that this accelerator always performs the predicated execution. Interestingly, the branch behavior of Xeon Phi falls between CPU and GPU. For the *PRT* matrix (Fig 11B), Xeon Phi performs the predicated execution similar to GPU, whereas the branch prediction clearly helps reduce the execution time on Xeon Phi for the *KDE* matrix when the KDE elements are sorted (Fig 11E). Nonetheless, the performance improvement for Xeon Phi is not as large as that for CPU because its computing cores are simpler and the wider SIMD vectors are generally less suitable for irregular data.

The original code improves the performance of computing *PRT* and *KDE*, however, it negatively impacts the calculation of the *MCS*. This effect can be attributed to the irregularity and shape of the *MCS* data structure containing a dense ligand_ColumnVector_, but a sparse MCS_Matrix_. Note that since protein_ColumnVector_ (Fig 11A–11C) and KDE_ColumnVector_ (Fig 11D–11F) data structures are 1D arrays, there is a branch pattern between different elements, which can be further improved by data sorting. This pattern is lost in the sparse MCS_Matrix_ × ligand_ColumnVector_ causing a significant branch prediction penalty and longer execution times for CPU and Xeon Phi (Fig 11G and 11H). On the GPU platform, we analyzed two versions of the generated Streaming ASSembly (SASS) code. The original SASS code always performs predicated execution, while the regulated SASS code uses non-predicated instructions without testing branch conditions. For that reason, the regulated docking code performs better for the irregular *MCS* data.

As mentioned above, the correlation between the computing time and the size of the *MCS* matrix also tends to be weaker than that for *PRT* and *KDE* matrices. For instance, the *R*^2^ for the original (regulated) code shown in blue (red) in Fig 11G–11I is 0.957 (0.946) for CPU, 0.720 (0.744) for Xeon Phi, and 0.793 (0.749) for GPU. This effect can be explained by the fact that the *MCS* data matrix is limited by the number of ligand atoms, which is between 6 and 62 for the CCDC/Astex dataset (Fig 6B). Consequently, the *MCS* matrix is not wide enough to efficiently utilize vector lanes on CPU (8 elements) and on Xeon Phi (16 elements) as well as the *x*-dimension of 2D CUDA thread blocks on GPU (32 elements); see Table 2. Consider a ratio of the data size and the number of cycles:

ratio=data_size_x/cycles Eq. 2

with the number of cycles required to traverse the *x*-dimension of the *MCS* matrix given by:

cycles=ceiling(data_size_x/vector_width_x) Eq. 3

For *PRT* and *KDE* matrices, whose data size is much larger than the vector width, the ratio in Eq 2 is close to the vector width yielding a strong linear correlation between the computing time and data size. In contrast, performance fluctuations caused by idle cycles created by the underutilized vector lanes (Eq 3) slightly decrease the correlation for the *MCS* matrix.

**Fig 12:**
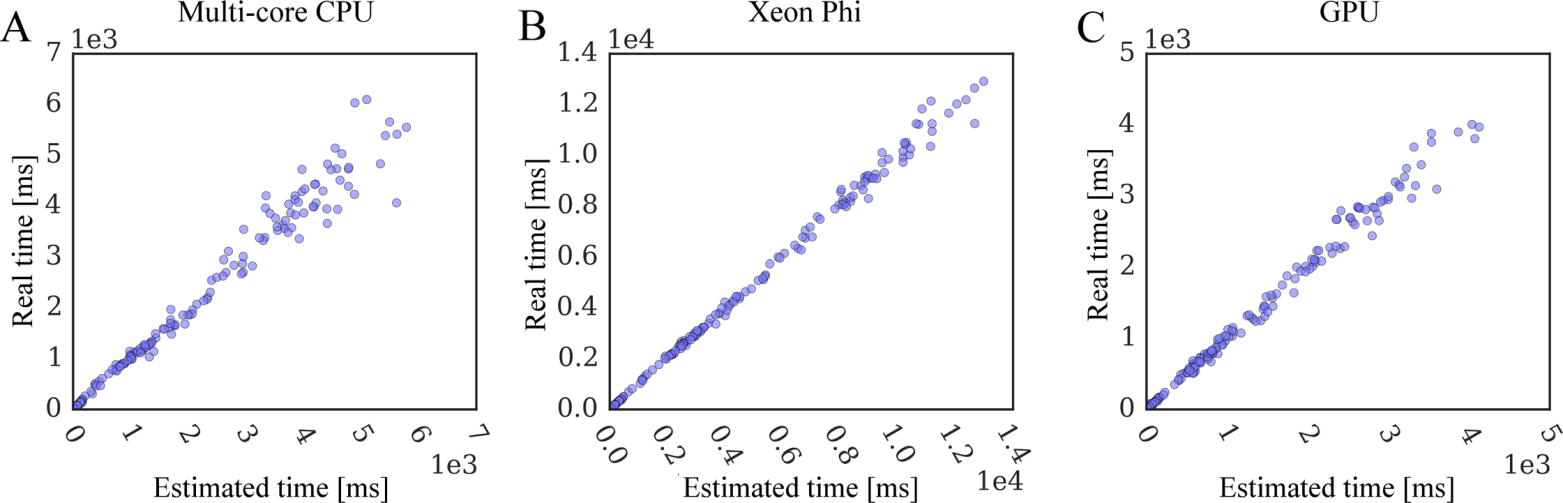
Correlation between the estimated and real docking time. Simulation time is estimated from static data size using a general linear regression model for (**A**) multi-core CPU, (**B**) Xeon Phi, and (**C**) GPU.

Encouragingly, the time required to compute various interaction matrices scales linearly with the static data size. Therefore, we developed the following general linear regression model to estimate the wall clock time for the docking kernel:

$$time\;=w_{1}PL+w_{2}KL+w_{3}ML+\;c$$ Eq. 4

where, *PL*, *KL*, and *ML* are the sizes of *PRT*, *KDE*, and *MCS* matrices, respectively. The fitted weights and the intercept (*w*_1_/*w*_2_/*w*_3_/*c*) are 7.493e-5/6.213e-6/5.121e-7/-0.025 for CPU, 2.343e-5/2.230e-6/5.937e-6/0.042 for Xeon Phi, and 4.798e-6/4.691e-6/1.783e-6/0.222 for GPU. Fig 12 shows that this model allows us to accurately predict the docking time from input data with the *R*^2^ of 0.974 on CPU (Fig 12A), 0.994 on Xeon Phi (Fig 12B), and 0.980 on GPU (Fig 12C). For those docking cases providing insufficient coarse-grained parallelism, we can further combine this linear regression with the performance model for the coarse-grained scaling (Fig 10). Specifically, the linear model predicts the average computing time for individual replicas assuming a sufficient coarse-grained parallelism. Since this value corresponds to the height of the first horizontal bar in Fig 10, we can estimate the execution time of a real task using the number of replicas and the repeating pattern of the regulated code.

### Comparative benchmarks of high-performance computing platforms

**Fig 13:**
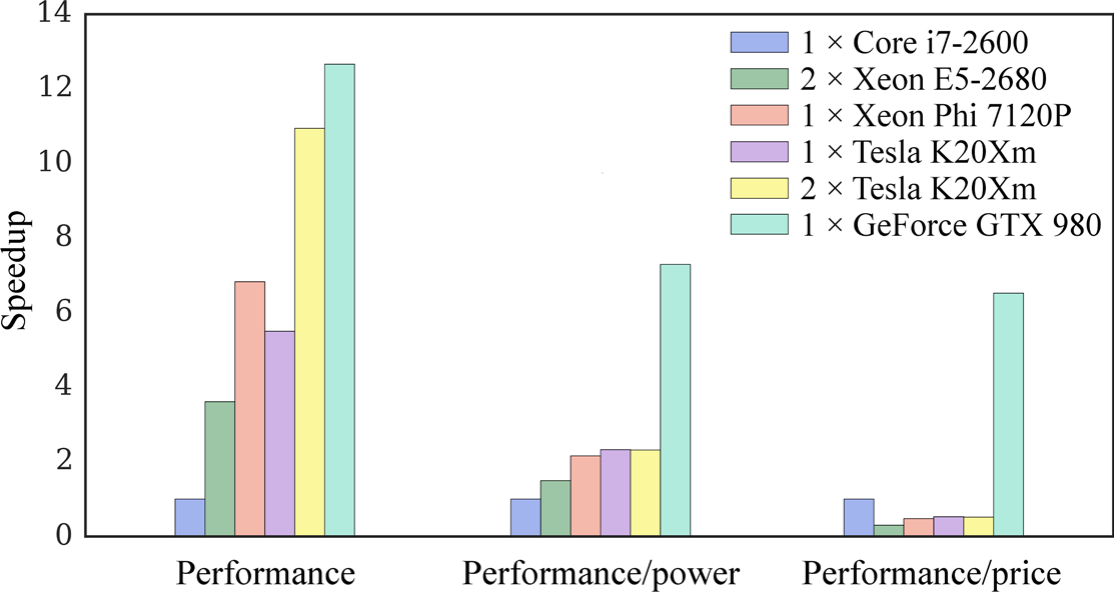
Benchmarks of GeauxDock against the CCDC/Astex dataset. Three measures are included, a pure computational performance, the performance divided by the energy consumption, and the performance divided by the hardware cost. Measurements for different platforms are normalized by the performance of Core i7-2600 CPU.

Finally, we perform comparative benchmarks of all computing platforms listed in Table 3 using the 1a07 target protein and the dataset of 204 CCDC/Astex ligands. In these simulations, we use the original GeauxDock code and the real data with respect to the number of protein and ligand conformations. Timing reports include the total execution time of the docking kernel for 204 tasks and the simulation wall time averaged over 8 independent docking runs for each task. GeauxDock is specifically designed for virtual screening applications, therefore, it reads the target protein input data only once for a given set of docking ligands. Indeed, GeauxDock spends from 95.4% (GeForce GTX 980) to 99.7% (Xeon E5-2680 v2) of the total time executing docking kernels, while loading and pre-processing input data take only about 10 seconds on average (Table 5). The reference time required to complete docking calculations for the entire dataset is 61.31 minutes using a multi-threaded CPU version running on Core i7-2600 multi-core CPU (platform D1, Table 3). Fig 13 shows that high-performance servers and hardware accelerators yield significant speedups over a mainstream PC desktop. GeForce GTX 980 is the fastest computing device in our tests, which achieves a 12.6× speedup and dramatically reduces the wall time to only 4.84 minutes. Xeon Phi gives a 6.8× speedup corresponding to the wall time of 9.00 minutes, whereas the performance of a single Tesla K20Xm card with 11.14 minutes of wall time is about 23% worse than Xeon Phi. It is noteworthy that we obtained almost a perfect scaling on multiple GPU cards; using a pair of K20Xm GPUs increases the performance by 98%, compared with a single K20Xm GPU. A dual Xeon E5-2680 CPU needs 16.99 minutes to complete docking calculations, which is about 3.6× faster than the baseline i7-2600 CPU running at a higher clock rate.

**Table 5:** Benchmarking data for docking simulations conducted for the CCDC/Astex dataset using various computing devices.

Computing device	Total wall time (kernel time) [min]	Theoretical peak performance [GFLOPS]	Power consumption [watt]	Price [US dollar]
1 × Core i7-2600 (platform D1)	61.31 (61.15)	224	95	283
2 × Xeon E5-2680 v2 (platform C1)	16.99 (16.86)	992	230	3440
1 × Xeon Phi 71200P (platform C1)	9.00 (8.79)	2553*	300	4129
1 × Tesla K20Xm (platform C1)	11.14 (11.01)	3936*	225	3000
2 × Tesla K20Xm (platform C2)	5.61 (5.46)	7872*	450	6000
1 × GeForce GTX 980 (platform D2)	4.84 (4.62)	4980*	165	550

Theoretical peak performance is taken from vendor specifications (Intel for CPU and Xeon Phi, and NVIDIA for GPU). GFLOPS stands for one billion single-precision floating-point operations per second. For devices supporting hardware fused multiply-add (FMA) instructions (marked with asterisks), 2 floating point operations for every FMA instruction are used to calculate GFLOPS. Estimated power consumption is taken from the thermal design power (TDP) document provided by the vendor. Price is the retail price suggested by the vendor at the time of the product release.

One should keep in mind that not only the theoretical peak performance, but also the cost and the energy consumption vary greatly for the testing platforms, particularly between consumer and server grade hardware (Table 5). For instance, a single Core i7 2600 is 12× less expensive and requires 59% less energy than a dual Xeon E5-2680 CPU, whereas GeForce GTX 980 is more than 5× lower priced and requires 27% less energy than Tesla K20Xm. For that reason, in addition to evaluating a pure computational performance, we analyze the performance with respect to the energy consumption and hardware cost. GeForce GTX 980 systematically outperforms other computing platforms, for example, it gives a benefit of 6.5× per dollar and 7.3× per watt compared to the reference D1 platform (Fig 13). This remarkable performance results from mapping massively parallel computations and data structure to the GPU architecture. According to vendor specifications, GeForce GTX 980 has a higher core utilization and better energy efficiency than the previous generation Tesla K20Xm. Its streaming multiprocessors have two-thirds of the number of scalar processors of Tesla K20Xm, yet the number of registers is the same. Moreover, the size of the shared memory on GeForce GTX 980 is twice as large as that on Tesla K20Xm. Therefore, extra efforts were devoted to tune the CUDA docking kernel in order to take advantage of the abundant resources on GeForce GTX 980. The performance per dollar of K20Xm GPU is comparable to a server grade Xeon E5-2680 CPU and Xeon Phi 7120P, but it is 2× lower than a consumer grade Core i7 processor. Due to advances in the semiconductor technology constantly improving the energy efficiency, the performance per watt of a server grade hardware (Xeon E5 CPU, Xeon Phi and K20Xm) is about twice as high as that for an inexpensive, yet two years older Core i7 processor.

### Case study

**Fig 14:**
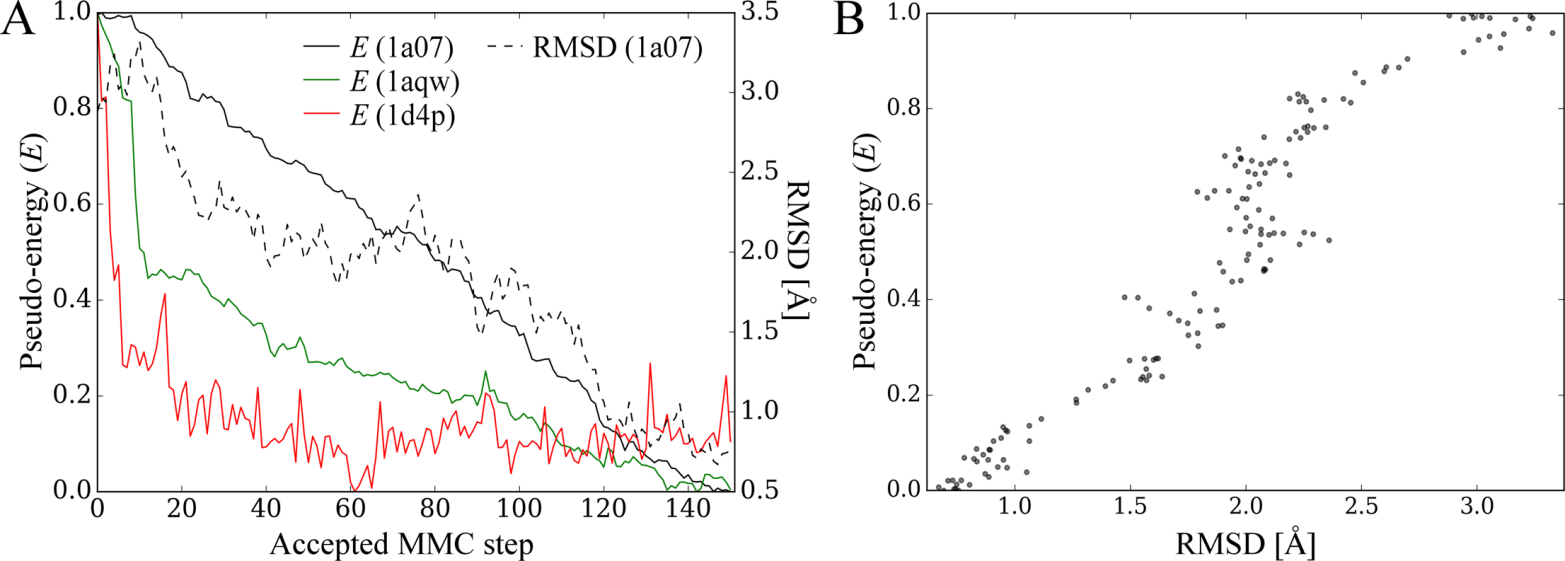
Examples of docking calculations using GeauxDock. Three cases are presented, a peptide ligand and C-src tyrosine kinase (PDB-ID: 1a07, black), glutathione and glutathione S-transferase (PDB-ID: 1aqw, green), as well as LY178550 and human α-thrombin (PDB-ID: 1d4p, red). (**A**) Solid lines show the pseudo-energy plotted as a function of the accepted Metropolis Monte Carlo (MMC) step; a trajectory of the RMSD is plotted for 1a07 (dashed black line). (**B**) Scatter plot of the RMSD and pseudo-energy for 1a07.

To demonstrate how GeauxDock samples the conformational space when searching for native conformations, in Fig 14, we present docking trajectories for several representative examples. In addition to the target complex 1a07 used in the profiling and benchmarking of parallel GeauxDock, we performed docking simulations of glutathione to glutathione S-transferase (PDB-ID: 1aqw) [40], and a non-peptidyl, active site-directed inhibitor LY178550 to human α-thrombin (PDB-ID: 1d4p) [41]. Docking ligands were initialized at random orientations within target binding pockets to mimic a real application, where the native conformations are unknown. Solid lines in Fig 14A show the trajectories of the pseudo-energy *E*_1_, *E*_2_ and *E*_3_ for 1a07, 1aqw and 1d4p, respectively. In all cases, the MMC sampling reached low-energy states with the fastest convergence for *E*_3_. On the other hand, pseudo-energy variations for *E*_1_ and *E*_2_ are smaller compared to *E*_3_, suggesting that the underlying energy surfaces for 1aqw and 1d4p are smoother.

In general, the convergence of molecular docking simulations is complicated by the fact that a large fraction of the search space may be sterically forbidden [13] and sophisticated scoring functions are often too sensitive to conformational changes in the binding regions [42]. To further investigate docking trajectories, we calculated the RMSD for each accepted MMC step during the docking process of 1a07. Encouragingly, the dashed black line in Fig 14A shows that the RMSD decreases with the decreasing pseudo-energy owing to the fact that both quantities are strongly correlated (Fig 14B). Altogether, these results demonstrate that the scoring function in GeauxDock effectively drives docking simulations toward native-like conformations.

### Comparison with other docking software

**Fig 15:**
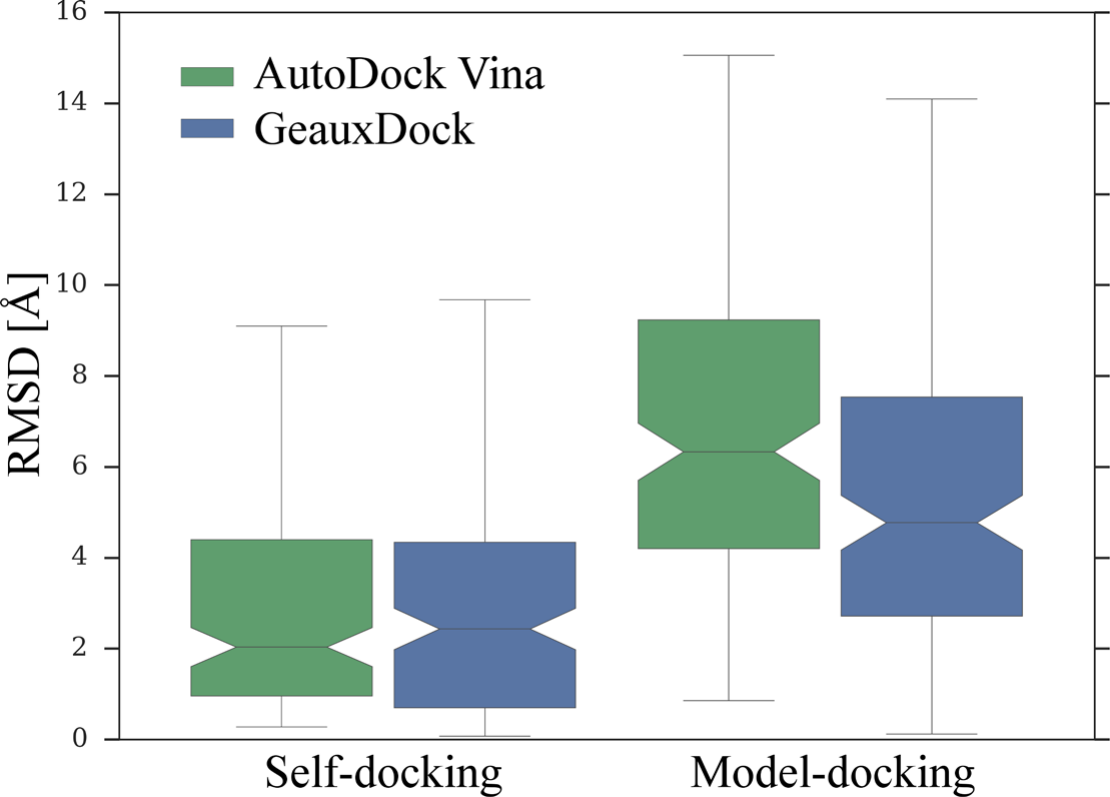
Docking accuracy of AutoDock Vina and GeauxDock on the PDBbind dataset. The performance is assessed by ligand heavy-atom RMSD calculated against experimental binding poses. A horizontal line inside each box is the median, boxes end at the first and the last quartile, and the whiskers span the distribution range of 10–90%. Two boxes on the left correspond to the self-docking experiment, whereas two boxes on the right are calculated for docking benchmarks against homology models.

Finally, in order to compare the docking accuracy of GeauxDock to the state-of-the-art, we performed benchmarking calculations of GeauxDock and AutoDock Vina [22] against the PDBbind dataset [43]. Here, we selected a set of 158 proteins whose length is below 600 residues. We ran both programs with the default parameters using randomized starting conformations of the docking ligands. The docking box for Vina was set to an optimal size based on the radius of gyration of query compounds, which was demonstrated to maximize docking accuracy [44]. First, we carried out a classical self-docking experiment, where the ligand is re-docked to the experimental protein structure co-crystalized with that compound. The geometric center of a ligand bound in the experimental complex structure was used as the binding pocket center for both programs. Docking accuracy is assessed by the RMSD calculated over ligand heavy atoms. Fig 15 (Self-docking) shows that the median ligand RMSD across the PDBbind dataset is 2.03 Å for Vina and 2.43 Å for GeauxDock. A *p*-value of 0.52 calculated by the Mann-Whitney U test demonstrates that the performance difference between Vina and GeauxDock in self-docking is statistically insignificant.

GeauxDock was designed to work with not only experimental structures, but also computer-generated models. Therefore, in addition to the self-docking experiment, we used both programs to dock ligands to the homology models of target proteins. Specifically, we constructed protein models for the PDBbind dataset using templates detected by HHsuite [45], whose sequence identity to the target is <70%. Moreover, in the model-docking experiment, we employed binding sites identified by *e*FindSite [28,36], so that ligand docking is performed solely with the predicted structural data. This dataset is clearly more challenging than that used in self-docking because of structural imperfections in the modeled target sites; the average heavy-atom RMSD calculated over binding site residues is 2.51 Å ±1.62. In addition, binding site locations are predicted with an average distance of 2.48 Å ±1.57 from the experimental pocket center. As expected, Fig 15 (Model-docking) shows that the median RMSD values for ligands docked by both programs tend to be higher than those obtained in the former experiment. Compared to self-docking, the median ligand RMSD for Vina increased by 4.30 Å to 6.33 Å. However, the median RMSD for GeauxDock is 4.77 Å, thus, it has increased only by 2.34 Å, a value that roughly corresponds to the structural distortions of target binding sites. Further, the *p*-value between both docking programs reported by the Mann-Whitney U test is now 0.00025 clearly demonstrating that GeauxDock significantly outperforms Vina in ligand docking against protein models.

## Conclusions

In this communication, we discuss the optimization of a molecular docking code, GeauxDock. GeauxDock features a novel scoring function and Monte Carlo-based conformational space sampling and it is designed for large-scale virtual screening applications using heterogeneous computer architectures. Because of its modular code framework, GeauxDock supports modern multi-core CPU, as well as Xeon Phi and GPU accelerators. Considerable efforts were devoted to minimize the data communication leading to at least 95% of the time spent on executing MMC kernels. Further, we applied various tuning techniques to significantly accelerate the docking kernel based on the performance characteristics obtained by a meticulous code profiling using diverse input data. For instance, a systematic optimization of the serial CPU code brought about not only a 6.5× speedup on a single computing core, but also a perfect scaling with the number of cores on modern shared-memory platforms equipped with multiple sockets of multi-core CPUs. Docking benchmarks conducted on many-core accelerators show that using Xeon Phi 7120P yields 1.9× performance improvement over a dual-socket Xeon E5 CPU, whereas the fastest GPU, GeForce GTX 980, achieves a 3.5× speedup over a dual CPU. It is important to note that in addition to hardware capabilities, a thorough code tuning for accelerator devices plays an important role in increasing the computational performance. For example, an early version of the GeauxDock code running on Tesla K20Xm was about 30% slower than a dual-socket Xeon E5 CPU, but after employing GPU intrinsic instructions, we were able to make K20Xm 53% faster. In addition to the evaluation of a purely computational performance, we examined the energy consumption and hardware costs. In conclusion, heterogeneous computing platforms, especially those equipped with the latest GPU cards, offer significant advantages over traditional CPU-based systems. Using parallel codes optimized for modern heterogeneous HPC architectures can significantly accelerate structure-based virtual screening applications. GeauxDock is open-sourced and publicly available from our website at www.brylinski.org/geauxdock and https://figshare.com/articles/geauxdock_tar_gz/3205249.

## Supporting Information

S1 Code Parallel execution of energy calculations in GeauxDock. The first section lists nine potentials included in the GeauxDock force field. Pseudo-codes for computations on protein_ColumnVector_ × ligand_RowVector_, KDE_ColumnVector_ × ligand_RowVector_, and MCS_Matrix_ × ligand_ColumnVector_ data structures are shown in the following sections.
(PDF)

S2 Code Data structures in GeauxDock. Data structures for (**A**) ligand conformation (first-level Structure of Arrays) and (**B**) ligand-protein complex (second-level Structure of Arrays).
(PDF)

S3 Code Strength reduction improving memory locality. An example of data structure and the corresponding computation (**A**) before and (**B**) after the strength reduction.
(PDF)

S4 Code Reduction of the arithmetic intensity. Part of the docking kernel (**A**) before and (**B**) after the strength reduction.
(PDF)

S5 Code Conformational sampling in GeauxDock. A pseudo-code for the Metropolis Monte Carlo algorithm used to sample the conformational space of protein-ligand complexes.
(PDF)
