# Supplementary material for: GeauxDock: Accelerating Structure-Based Virtual Screening with Heterogeneous Computing
Source: PLoS One. 2016 Jul 15;11(7):e0158898. doi: 10.1371/journal.pone.0158898 (PMC4946785; doi:10.1371/journal.pone.0158898)
Supplement: S1 Code — The first section lists nine potentials included in the GeauxDock force field. Pseudo-codes for computations on proteinColumnVector × ligandRowVector, KDEColumnVector × ligandRowVector, and MCSMatrix × ligandColumnVector data structures are shown in the following sections. (PDF) [file pone.0158898.s001.pdf]

Supporting Information for "GeauxDock: Accelerating structure-based virtual screening with heterogeneous computing"

S1 Code. Parallel execution of energy calculations in GeauxDock

Contact-dependent potentials
E_{CP} ← generic contact potential
E_{CP}^{PS} ← pocket-specific contact potential

Distance-dependent potentials
E_{ele}^{soft} ← soft electrostatics
E_{vdW}^{soft} ← soft van der Waals
E_{HB} ← hydrogen bonds
E_{HP} ← hydrophobic interactions

Other potentials
E_{KDE} ← pseudo-pharmacophore
E_{MCS} ← family-conserved anchor substructures
E_{DST} ← distance restraint

Computations on $\text{protein}_{\text{ColumnVector}} \times \text{ligand}_{\text{RowVector}}$ (PRT)

L ← number of ligand atoms
P ← number of protein effective points
D_{lp} ← distance between ligand atom *l* and protein point *p*
D_{lp}^{cnt} ← type-dependent distance threshold for ligand-protein contacts
energy[9] ← nine individual energy terms
energy_partial[5][*L*][*P*] ← partial results from the parallel execution
temp1[*L*][*P*], *temp2*[*L*] ← variables to store partial results

parallel for *l* = 0 to *l* < *L* **do**
  parallel for *p* = 0 to *p* < *P* **do**
    calculate E_{ele}^{soft}, E_{vdW}^{soft}, and E_{HB}, and store in *energy_partial*[0,1,2][*l*][*p*]
    if $(D_{lp} \le D_{lp}^{cnt})$ **then**
      calculate E_{CP} and E_{CP}^{PS}, and store in *energy_partial*[3,4][*l*][*p*]
      calculate E_{HP} and store in *temp1*[*l*][*p*]
    end if
  end for
  temp2[*l*] ← Σ_p^P *temp1*[*l*][*p*]
end for

for *i* = 0 to *i* < *5* **do**
  energy[*i*] ← $\Sigma_l^L \Sigma_p^P$ *energy_partial*[*i*][*l*][*p*]
end for
energy[*6*] ← Σ_l^L function (*temp2*[*l*])

Computations on $\text{KDE}_{\text{ColumnVector}} \times \text{ligand}_{\text{RowVector}}$ (KDE)

$K \leftarrow$ number of KDE points
$temp3[L][K]$, $temp4[L] \leftarrow$ variables to store partial results

parallel for $l = 0$ to $l < L$ **do**
parallel for $k = 0$ to $k < K$ **do**
if (l and k are of the same type) **then**
calculate E_{KDE} and store in $temp3[l][k]$
end if
end for
$temp4[l] \leftarrow \Sigma_k^K\ temp3[l][k]$
end for

energy[7] $\leftarrow \Sigma_l^L$ function ($temp4[l]$)

Computations on $\text{MCS}_{\text{Matrix}} \times \text{ligand}_{\text{ColumnVector}}$ (MCS)

$R \leftarrow$ number of rows in a sparse $\text{MCS}_{\text{Matrix}}$
$C \leftarrow$ number of columns in a sparse $\text{MCS}_{\text{Matrix}}$
$L \leftarrow$ number of ligand atoms
C equals L
$temp5[R][C]$, $temp6[R] \leftarrow$ variables to store partial results

parallel for $r = 0$ to $r < R$ **do**
parallel for $c = 0$ to $c < C$ **do**
if ($\text{MCS}_{\text{Matrix}}[r][c] \neq 0$) **then**
calculate E_{MCS} using $\text{MCS}_{\text{Matrix}}$ and $\text{ligand}_{\text{ColumnVector}}$, and store in $temp5[r][c]$
end if
end for
$temp6[r] \leftarrow \Sigma_c^C\ temp5[r][c]$
end for

energy[8] $\leftarrow \Sigma_r^R$ function($temp6[r]$)

energy[9] $\leftarrow$ calculate E_{DST}

Calculation of the total energy

$\alpha[9]$, $\beta \leftarrow$ parameters for the linear combination of individual energy terms

$total\_energy \leftarrow \Sigma_i^9\ (\alpha[i] \times energy[i]) + \beta$
