# Supplementary material for: GeauxDock: Accelerating Structure-Based Virtual Screening with Heterogeneous Computing
Source: PLoS One. 2016 Jul 15;11(7):e0158898. doi: 10.1371/journal.pone.0158898 (PMC4946785; doi:10.1371/journal.pone.0158898)
Supplement: S2 Code — Data structures for (A) ligand conformation (first-level Structure of Arrays) and (B) ligand-protein complex (second-level Structure of Arrays). (PDF) [file pone.0158898.s002.pdf]

Supporting Information for "GeauxDock: Accelerating structure-based virtual screening with heterogeneous computing"

S2 Code A. Data structure for a ligand conformation (first-level Structure of Arrays)

```
struct Ligandconf {
  float x[L]; // x-coordinate
  float y[L]; // y-coordinate
  float z[L]; // z-coordinate
  int t[L]; // atom type
  float c[L]; // electric charge
  int center_xyz[3]; // geometric center
  int atom_number; // number of atoms
};
```

S2 Code B. Data structure for a ligand-protein complex (second-level Structure of Arrays)

```
struct Complex {
  Ligandconf ligandconf [MAX_LIG_CONFORMATION_NUM];
  Proteinconf proteinconf [MAX_PRT_CONFORMATION_NUM];
  Temperature temperature[MAX_TEMP_NUM];
  KDE kde;
  MCS mcs;
  PSP psp;
  ReplicaInfo replica_info[MAX_REPLICA_NUM];
  ComplexSize complexsize;
};
```
