# Supplementary material for: GeauxDock: Accelerating Structure-Based Virtual Screening with Heterogeneous Computing
Source: PLoS One. 2016 Jul 15;11(7):e0158898. doi: 10.1371/journal.pone.0158898 (PMC4946785; doi:10.1371/journal.pone.0158898)
Supplement: S3 Code — An example of data structure and the corresponding computation (A) before and (B) after the strength reduction. (PDF) [file pone.0158898.s003.pdf]

Supporting Information for "GeauxDock: Accelerating structure-based virtual screening with heterogeneous computing"

S3 Code A. Example of a data structure and the corresponding computation before strength reduction

Data structure	Pre-processing	Docking kernel
struct **ProteinConf** {<br>int *r*[*P*];<br>int *seq3*[*P*];<br>...<br>}	none	int *r = prtconf.r[index];*<br>int *seq3 = prtconf.seq3[r];*

S3 Code B. Data structure and computation after strength reduction improving memory locality

Data structure	Pre-processing	Docking kernel
struct **ProteinConf** {<br>int *seq3r*[*P*];<br>...<br>}	*seq3r*[*i*] = *prtconf.seq3*[*prt.r*[*i*]]	int *seq3* = *prtconf.seq3r*[*index*];
