# Supplementary material for: GeauxDock: Accelerating Structure-Based Virtual Screening with Heterogeneous Computing
Source: PLoS One. 2016 Jul 15;11(7):e0158898. doi: 10.1371/journal.pone.0158898 (PMC4946785; doi:10.1371/journal.pone.0158898)
Supplement: S4 Code — Part of the docking kernel (A) before and (B) after the strength reduction. (PDF) [file pone.0158898.s004.pdf]

Supporting Information for "GeauxDock: Accelerating structure-based virtual screening with heterogeneous computing"

S4 Code A. Part of the docking kernel before the strength reduction

Pre-processing	Docking kernel (for computing the soft van der Waals potential)
none	float *r1* = *par.vdw*[*i*][*j*][0];<br>float *e1* = *par.vdw*[*i*][*j*][1];<br>float *p1* = (2.0 × *e1* × pow(*par.lj*[2] × *r1*, 9)) / (pow(*dst*, 9));<br>float *p2* = (3.0 × *e1* × pow (*par.lj*[2] × *r1*, 6)) / (*pow*(*dst*, 6));<br>float *p4* = *p1* × *par.lj*[0] × (1.0 + *par.lj*[1] × *pow*(*dst*, 2)) + 1.0f;<br>*evdw* += (*p1* – *p2*) / *p4*;

S4 Code B. Part of the docking kernel after the reduction of the arithmetic intensity

Pre-processing	Docking kernel (reused by various code blocks)
float *tmp* = *par.lj*[2] × *par.vdw*[*i*][*j*][0];<br>float *e1* = *par.vdw*[*i*][*j*][1];<br>*par.p1a*[i][j] = 2.0f × *e1* × powf(*tmp*, 9.0f);<br>*par.p2a*[i][j] = 3.0f × *e1* × powf(*tmp*, 6.0f);<br>*par_lj0* = *enepara_lj*[0];<br>*par_lj1* = *enepara_lj*[1];	float *dst_pow2* = *dst* × *dst*;<br>float *dst_pow4* = *dst_pow2* × *dst_pow2*<br><br>Docking kernel (for computing the soft van der Waals potential)<br><br>float *p1* = *par.p1a*[*i*][*j*] / (*dst_pow4* × *dst_pow4* × *dst*);<br>float *p2* = *par.p2a*[*i*][*j*] / (*dst_pow4* × *dst_pow2*);<br>float *p4* = *p1* × *par_lj0* × (1.0f + *par_lj1* × *dst_pow2*) + 1.0f;<br>*evdw* += (*p1* – *p2*) / *p4*;
