# Supplementary material for: GeauxDock: Accelerating Structure-Based Virtual Screening with Heterogeneous Computing
Source: PLoS One. 2016 Jul 15;11(7):e0158898. doi: 10.1371/journal.pone.0158898 (PMC4946785; doi:10.1371/journal.pone.0158898)
Supplement: S5 Code — A pseudo-code for the Metropolis Monte Carlo algorithm used to sample the conformational space of protein-ligand complexes. (PDF) [file pone.0158898.s005.pdf]

Supporting Information for "GeauxDock: Accelerating structure-based virtual screening with heterogeneous computing"

S5 Code. Conformational sampling with the Metropolis Monte Carlo algorithm

$cycle_{\text{max}}$ ← number of Monte Carlo cycles
C_{old} ← old configuration
E_{old} ← pseudo-energy of configuration C_{old}

for Monte Carlo cycle $c = 1$ to $cycle_{\text{max}}$ **do**
  C_{new} ← new configuration, generated by randomly perturbing C_{old}
    E_{new} ← pseudo-energy of configuration C_{new}
    $E_{\text{diff}} = E_{\text{new}} - E_{\text{old}}$
    $probability = \mathrm{e}^{E_{\text{diff}}/\text{temperature}}$
    r ← random number from 0 to 1
    if (($E_{\text{diff}} < 0$) or ($r < probability$)) **then**
      S_{old} ← S_{new}
      E_{old} ← E_{new}
    end if
end for
